# Identification of the metabolites of ivermectin in humans

**DOI:** 10.1002/prp2.712

**Published:** 2021-01-26

**Authors:** Phornpimon Tipthara, Kevin C. Kobylinski, Markus Godejohann, Borimas Hanboonkunupakarn, Alison Roth, John H. Adams, Nicholas J. White, Podjanee Jittamala, Nicholas P. J. Day, Joel Tarning

**Affiliations:** ^1^ Mahidol Oxford Tropical Medicine Research Unit Faculty of Tropical Medicine Mahidol University Bangkok Thailand; ^2^ Department of Entomology Armed Forces Research Institute of Medical Sciences Bangkok Thailand; ^3^ AIC Bruker BioSpin GmbH Rheinstetten Germany; ^4^ Department of Clinical Tropical Medicine Faculty of Tropical Medicine Mahidol University Bangkok Thailand; ^5^ Center for Global Health and Infectious Diseases Research College of Public Health University of South Florida Tampa FL USA; ^6^ Department of Drug Discovery Experimental Therapeutics Branch Walter Reed Army Institute of Research Silver Spring MD USA; ^7^ Centre for Tropical Medicine and Global Health Nuffield Department of Clinical Medicine University of Oxford Oxford United Kingdom; ^8^ Department of Tropical Hygiene Faculty of Tropical Medicine Mahidol University Bangkok Thailand

**Keywords:** ivermectin, LC‐MS/MS, malaria, metabolism

## Abstract

Mass drug administration of ivermectin has been proposed as a possible malaria elimination tool. Ivermectin exhibits a mosquito‐lethal effect well beyond its biological half‐life, suggesting the presence of active slowly eliminated metabolites. Human liver microsomes, primary human hepatocytes, and whole blood from healthy volunteers given oral ivermectin were used to identify ivermectin metabolites by ultra‐high performance liquid chromatography coupled with high‐resolution mass spectrometry. The molecular structures of metabolites were determined by mass spectrometry and verified by nuclear magnetic resonance. Pure cytochrome P450 enzyme isoforms were used to elucidate the metabolic pathways. Thirteen different metabolites (M1‐M13) were identified after incubation of ivermectin with human liver microsomes. Three (M1, M3, and M6) were the major metabolites found in microsomes, hepatocytes, and blood from volunteers after oral ivermectin administration. The chemical structure, defined by LC‐MS/MS and NMR, indicated that M1 is 3″‐*O*‐demethyl ivermectin, M3 is 4‐hydroxymethyl ivermectin, and M6 is 3″‐*O*‐demethyl, 4‐hydroxymethyl ivermectin. Metabolic pathway evaluations with characterized cytochrome P450 enzymes showed that M1, M3, and M6 were produced primarily by CYP3A4, and that M1 was also produced to a small extent by CYP3A5. Demethylated (M1) and hydroxylated (M3) ivermectin were the main human *in vivo* metabolites. Further studies are needed to characterize the pharmacokinetic properties and mosquito‐lethal activity of these metabolites.

## Graphical abstract

Thirteen different phase 1 metabolites of ivermectin (M1‐M13) were identified using pooled human liver microsomes. Demethylation (M1) and hydroxylation (M3) of ivermectin were demonstrated to be the two main metabolism pathways in human volunteers.

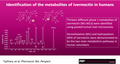

**Abbreviations**
CYP450: cytochrome P450
HLM: human liver microsomes
HMBC: heteronuclear multiple bond correlation
HRMS: high‐resolution mass spectrometry
HSQC: heteronuclear single quantum correlation
IVM: ivermectin
MDA: mass drug administration
NADPH: reduced form of nicotinamide adenine dinucleotide phosphate
NMR: nuclear magnetic resonance
NTDs: neglected tropical diseases
PHH: primary human hepatocytes
UHPLC‐Q‐TOF‐MS: ultra‐high performance liquid chromatography quadrupole time‐of‐flight mass spectrometry

## 1 INTRODUCTION

Ivermectin (IVM) is an antiparasitic and endectocidic drug used for decades in animal health and for treating onchocerciasis, lymphatic filariasis, scabies, and strongyloidiasis in humans.^1^ IVM also has some antiviral activity including against SARS‐CoV‐2 *in vitro*.^2^

Malaria is a mosquito‐borne disease transmitted by *Anopheles* mosquitoes during blood feeding. Numerous studies have reported the mosquito‐lethal effect of IVM^3^, ^4^, ^5^ and the ability to inhibit sporogony of *Plasmodium* in the mosquito.^6^, ^7^, ^8^ Mass drug administration (MDA) of IVM has been suggested as a possible vector control tool to aid malaria elimination as it has been shown to reduce *Plasmodium* transmission by mosquitoes^9^ and reduce transmission to humans.^10^ A recent clinical trial in Thailand showed that mosquito‐lethal effects persisted well beyond the detectable presence of the parent compound, which suggests that IVM may have active metabolites that are more slowly eliminated than the parent compound.^5^

**FIGURE 1:**
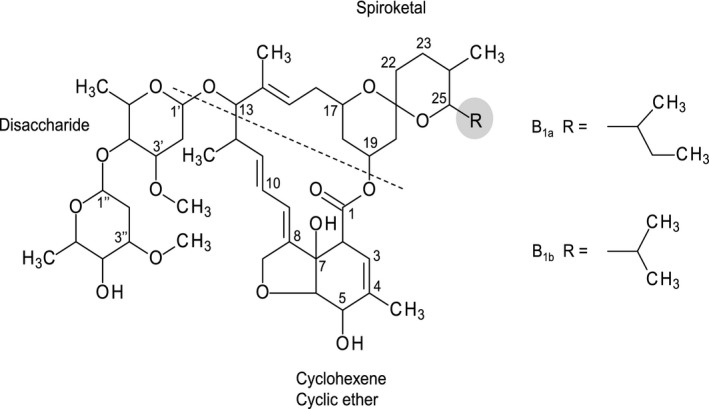
Molecular structure of ivermectin. IVM consists of a spiroketal unit (C17–C28), cyclohexene cyclic ether unit (C2–C8), and a disaccharide unit at C13. A secondary butyl side chain at C25 give rise to the major component (IVM‐B_1a_) and an isopropyl side chain give rise to the minor component (<10%; IVM‐B_1b_)

IVM is a semisynthetic compound derived from avermectin (B_1_ series), a natural fermentation product of the soil bacterium *Streptomyces avermitilis*. The regiospecific hydrogenation of avermectin B_1_ at the 22,23‐double bond produces the 22,23‐single bond derivative called 22,23‐dihydroavermectin B_1_ or IVM.^11^ IVM is a mixture containing at least 90% of 22,23‐dihydroavermectin B_1a_ (H_2_B_1a_ or IVM‐B_1a_) and less than 10% of 22,23‐dihydroavermectin B_1b_ (H_2_B_1b_ or IVM‐B_1b_). The chemical structures show an alkyl side chain difference between IVM‐B_1a_ and IVM‐B_1b_ at C25 (Figure 1). Both compounds have the same antiparasitic activity.^12^

There have been several studies of IVM metabolites produced in nonhuman vertebrates.^13^, ^14^, ^15^, ^16^, ^17^ The major metabolite found in rats, cattle, and sheep is the 24‐hydroxymethyl derivative^13^, ^16^ while only trace levels are found in pigs. The 3″‐*O*‐demethyl derivative is the major metabolite present in pigs.^14^ A previous *in vitro* study using human liver microsomes found nine IVM metabolites, mostly hydroxylated and demethylated compounds including the two listed above.^18^

In this study, we aimed to identify the common metabolites in humans through *in vitro* and *in vivo* experiments. Pooled human liver microsomes and primary human hepatocytes were exposed to IVM, and metabolite fractions were collected to identify metabolites produced *in vitro*. Human whole blood, collected from healthy volunteers after a single oral dose of IVM (400 µg/kg), was used to identify metabolites produced *in vivo*. While the standard dose of ivermectin is 200 µg/kg, the 400 µg/kg dose has been shown to be safe and efficacious in numerous clinical trials, is frequently used in clinical settings, and is now recommended on the package insert for lymphatic filariasis mass drug administration. Previous pharmacokinetic modeling and *in vitro* mosquito mortality experiments indicated that IVM 400 µg/kg is the ideal dose to target the important *Anopheles* malaria vectors in the Greater Mekong Subregion.^8^ The structure of IVM metabolites were characterized by LC–MS/MS and verified by NMR. The metabolic pathways that generated these metabolites were characterized by incubation of IVM with purified human cytochrome P450 (CYP) enzymes, followed by LC–MS/MS analysis.

## 2 MATERIALS AND METHODS

### 2.1 Chemicals

IVM (IVM‐B_1a_ > 95%, IVM‐B_1b_ < 2%) was purchased from Sigma‐Aldrich. IVM‐B_1a_ (95% purity) was purchased from Toronto Research Chemicals Inc. IVM‐B_1b_ (99.27% purity) was purchased from Clearsynth Labs Ltd. Pooled human liver microsomes (50 donors, Gibco™) were purchased from Thermo Fisher Scientific. Cryopreserved primary human hepatocytes (M00995‐P, UBV donor) and hepatocyte culture medium (InVitroGRO™ CP Medium) were purchased from BioIVT LLC. cDNA‐expressed human CYP isoenzymes 1A2, 2B6, 2C8, 2C9, 2C18, 2C19, 2D6, 2E1, 3A4, and 3A5 (Corning^®^ Supersomes™), insect cell control microsomes, human P450 cytochrome *b_5_* oxidoreductase, and NADPH regenerating system were purchased from Corning Inc. LC–MS grade water and acetonitrile were purchased from J.T Baker. LC–MS grade ammonium acetate, formic acid, and HPLC grade organic solvent for NMR analysis were purchased from Sigma‐Aldrich. CD_3_CN 99.8% and DCOOD 98% were purchased from Deutero GmbH. Water for NMR was prepared from a Milli‐Q purification system from Merck.

### 2.2 Pooled human liver microsomes

Pooled human liver microsomes (containing 20 mg/ml of protein) were thawed on ice. Microsome reactions were performed in microcentrifuge tubes by adding 183 µl of 0.1 M potassium phosphate buffer (pH 7.4), 2 µl of 1.0 mM IVM (prepared in acetonitrile 80% (v/v)), and 5 µl of microsomes. The tube was vortexed briefly and incubated at 37°C for 5 min in a shaking water bath. The reaction was initiated by adding 10 µl of 20 mM NADPH (reduced form of nicotinamide adenine dinucleotide phosphate) prepared in 100 mM potassium phosphate buffer pH 7.4. Total reaction volume per tube was 200 µl with the final concentration of 10 µM IVM and 1.0 mM NADPH. Each tube was vortexed briefly and a baseline sample (0 min control) was collected by aliquoting 100 µl of the metabolite fraction mixture described above to a separate tube with 100 µl of pre‐chilled acetonitrile, which was kept on ice until centrifugation. Two separate negative control tubes were prepared, one without NADPH (negative co‐factor control) but with an extra 10 µl of buffer and a second tube without IVM substrate (negative IVM control) with an extra 2 µl of buffer. All remaining reactions, including negative co‐factor control, and negative IVM control, were incubated at 37°C for 60 min with gentle shaking. After 60 min of incubation, all tubes were removed from the water bath and cold acetonitrile was added immediately to make a final 1:1 (v/v) ratio. All tubes (0‐ and 60‐min reactions, negative co‐factor, and negative IVM controls) were vortexed briefly again and centrifuged at 10,000*g* for 15 min at 4°C. The supernatant was collected and kept frozen at −80°C until LC–MS/MS analysis. The reactions described above were performed using IVM, pure IVM‐B_1a_ and pure IVM‐B_1b_ as substrates (biological triplicate incubations for each substrate).

For NMR analysis, 60 tubes were prepared each with 10 µl of 1.0 mM IVM, 915 µl of 100 mM potassium phosphate buffer (pH 7.4), 25 µl of microsomes, and 50 µl of 20 mM NADPH for a final volume of 1 ml. Reactions were stopped at 60 min with ice‐cold acetonitrile and centrifuged immediately as describe above. The supernatant was collected, evaporated in speed vacuum, and kept frozen at −80°C until NMR analysis.

### 2.3 Primary human hepatocytes

Primary human hepatocytes were seeded on a 384‐well plate as described previously to stimulate reacquisition of *in vivo* physiologic activity.^19^ At day 3 post seed, IVM (10 µM) was added to each well. There were no media changes and no subsequent additions of IVM. Forty microliters of media was collected from five individual wells at 24 hours and pooled in a microcentrifuge tube (total volume 200 µl). Forty microliters of media was mixed with cold acetonitrile (160 µl), vortexed for 10 min at ambient temperature, and centrifuged at 10,000*g* for 15 min at 4°C (triplicate extractions). The supernatant was collected, evaporated in speed vacuum, and kept frozen at −80°C until LC–MS/MS analysis.

### 2.4 Healthy volunteer samples

Venous blood was collected from three healthy Thai volunteers given a single oral dose of IVM (400 µg/kg) (NCT02568098).^5^ The blood samples were collected in sodium heparin tubes at 24 h post‐IVM ingestion and kept frozen at −80°C until LC–MS/MS analysis. For metabolite extraction, whole blood samples were thawed at ambient temperature, vortexed briefly, and centrifuged at 3000*g* for 5 min at 20°C. Fifty microliters of plasma was transferred into a new microcentrifuge tube and mixed with 200 µl of cold acetonitrile. Tubes were vortexed for 10 min at ambient temperature and centrifuged at 1100*g* for 5 min at 20°C. The supernatant was collected, evaporated in speed vacuum, and kept at −80°C freezer until LC–MS/MS analysis.

### 2.5 cDNA‐expressed cytochrome P450 enzyme

The metabolism of IVM was studied *in vitro* using cDNA‐expressed human CYP isoenzymes 1A2, 2B6, 2C8, 2C9, 2C18, 2C19, 2D6, 2E1, 3A4, 3A5 (1.0 µM). In separate tubes for each CYP isoform, the following reagents were added: 166 µl of 0.1 M potassium phosphate buffer (pH 7.4), 2 µl of 1.0 mM IVM (prepared in acetonitrile 80% (v/v)), and 20 µl of CYP enzyme. A separate tube was prepared for the negative control (no human CYP enzyme) by adding 20 µl of insect cell control microsomes. A second tube of negative control was prepared, to evaluate the effect of reductase, by adding 20 µl of human P450 cytochrome *b_5_* oxidoreductase. The tubes were vortexed briefly and incubated at 37°C for 5 min in a shaking water bath. The reactions were initiated by adding 12 µl of NADPH regenerating system solution (premixed 10 µl of solution A and 2 µl of solution B). Each tube was vortexed briefly and a baseline sample (0 min control) was collected by aliquoting 100 µl of the incubation to a separate tube with 100 µl of prechilled acetonitrile, which was held on ice until centrifugation. All tubes (reactions and controls) were incubated at 37°C for 60 min with gentle shaking. After 60 min of incubation, all tubes were removed from the water bath and cold acetonitrile was added immediately at a 1:1 ratio (v/v). Tubes were vortexed briefly and centrifuged at 10,000*g* for 15 min at 4°C. The clear supernatant was collected into new microcentrifuge tubes and kept frozen at −80°C until LC–MS/MS analysis. The incubations and reactions described above were performed in triplicate.

### 2.6 UHPLC‐Q‐TOF‐MS

The LC–MS/MS system used was an ultra‐high performance liquid chromatography (Agilent 1260 Quaternary pump, Agilent 1260 High Performance autosampler, and Agilent 1290 Thermostatted Column Compartment SL, Agilent Technologies) coupled to a quadrupole time‐of‐flight mass spectrometer (Q‐TOF‐MS) (TripleTOF 5600^+^, Sciex) with an electrospray ionization (ESI) using a DuoSpray ion source. The mobile phase system for UHPLC was water containing 10 mM ammonium acetate and 0.1% formic acid (mobile phase A) and acetonitrile:water at a 95:5 ratio (v/v) containing 10 mM ammonium acetate and 0.1% formic acid (mobile phase B). Human liver microsome extracts were transferred directly to a LC vial for injection. Evaporated supernatant from primary human hepatocytes and whole blood samples were reconstituted in 20 and 50 μl, respectively, of mobile phase at starting gradient (mobile phase A:B at a ratio of 60:40 (v/v)) and transferred to a LC vial for injection. LC vials were kept in the autosampler at 6°C during analysis. Five microliters of sample was injected on to a C18 reversed‐phase column (ACQUITY UPLC HSS T3, 2.1 × 100 mm, 1.8 µm; Waters Corporation) protected by a precolumn (ACQUITY UPLC HSS T3, 2.1 × 5 mm, 1.8 µm, Waters), for separation by UHPLC at a flow rate of 0.3 ml/min at 40°C. The UHPLC elution gradient was started at 40% mobile phase B for 2.0 min (0–2.0 min), followed by 40%–80% B for 2.0 min (2.0–4.0 min), 80%–100% B for 5.0 min (4.0–9.0 min), 100% B for 5.0 min (9.0–14.0 min), 100%–40% B for 0.1 min (14.0–14.1), and 40% B for 3.9 min (14.1–18.0 min). The UHPLC‐Q‐TOF‐MS system, mass ion chromatogram, and mass spectra were acquired by Analyst™ Software version 1.7 (SCIEX). The Q‐TOF‐MS was operated in ESI‐positive mode at ion source gas 1 (GS1) of 40 psi, ion source gas 2 (GS2) of 40 psi, curtain gas (CUR) of 30 psi, ion spray voltage floating (ISVF) of 4500 V, source temperature (TEM) at 350°C, and declustering potential (DP) of 120 V. Data were acquired in the informative‐dependent acquisition (IDA) mode composed of a TOF‐MS scan and 10 dependent product ion scans in the high sensitivity mode with dynamic background subtraction. Mass range of TOF‐MS scan was at *m*/*z* 100–1000 and product ion scan was at *m*/*z* 50–1000. IVM standard solution (100 ng/ml) was injected before and after batch analysis for validating the system performance.

### 2.7 LC‐SPE‐NMR/MS

The analyses were performed by in‐line instruments of interfacing liquid chromatography with parallel NMR and mass spectrometry. The 60 dried microsome pellets described above were each reconstituted in 300 µl of methanol and sonicated for 5 min. Supernatants were pooled into one 50 ml falcon tube. A second extraction of the microsome residue was performed by adding an additional 300 µl of acetonitrile followed by sonication for 5 min. The supernatant from the second extraction was then transferred to the same 50 ml falcon tube described above. The pooled supernatant was evaporated under nitrogen gas to a final volume of 200 µl and transferred to an HPLC vial. The extract (33 µl) was injected into the HPLC (Agilent 1260) at a flow rate of 0.5 ml/min at 25°C on a 250 × 4.6 mm, 5 µm Kinetex EVO C18 column (Phenomenex). Mobile phase A consisted of water with 0.1% formic acid‐d_2_ (DCOOD) and mobile phase B consisted of acetonitrile with 0.1% DCOOD. The elution gradient was started at 50% mobile phase B for 2.0 min (0–2.0 min), followed by a linear increase from 50% B to 100% B over the next 33.0 min (2.0–35.0 min), ending on 100% B for 5.0 min (35.0–40.0 min). UV detection was done at 240 nm. An MS Bridge interface (Bruker Biospin) was used to split a small portion of the effluent from the HPLC column and direct it to the ion source of a MicrOTOF‐QII mass spectrometer (Bruker Daltonik, Bremen, Germany) using an acetonitrile makeup flow of 70 µl/min. The mass spectrometer was operated in the positive ionization mode with a scan range at *m*/*z* 50 to 1000. Mass calibration was done with sodium acetate infused at the beginning of the chromatography. The isolated metabolites were trapped postcolumn on 2 × 10 mm solid phase extraction cartridges filled with HySphere GP resin using the Prospekt 2 SPE interface from Spark Holland. The peaks of three injections (each 33 µl) were combined on individual cartridges (multi trapping). In total, 2 × 3 trappings were performed. The makeup flow rate for the trapping was 1.5 ml/min. After chromatography the cartridges including the metabolites were dried with nitrogen gas and the two cartridges containing the metabolites were eluted with each 300 µl of acetonitrile‐d_3_ (CD_3_CN) into 5‐mm NMR tubes. In total, six trappings were finally transferred to the NMR tube for each metabolite resulting in a total volume of 600 µl. The API reference sample was measured with a 500 MHz AVANCE III NMR spectrometer equipped with a nitrogen cooled 5‐mm Prodigy TCI (triple resonance inverse configuration of the coils with a cooled carbon channel) cryoprobe. Postcolumn SPE fractions of the metabolites were measured first with the 500 MHz spectrometer and later with an 800 MHz Neo NMR spectrometer equipped with a helium cooled 5‐mm TCI cryoprobe (Bruker Biospin). SPE fractions were analyzed with the CMC‐se software using optimized parameter sets including the Proton‐1D, edited heteronuclear single coherence spectroscopy (HSQC) and heteronuclear multiple bond coherence spectroscopy (HMBC). In addition, selective HMBC experiments were performed for areas of closely resonating carbon resonances. IVM (5.2 mg) was dissolved in deuterated acetonitrile (1 ml) and transferred to a 5‐mm NMR tube and run as a reference compound.

### 2.8 Data analysis

Metabolite identification was done by MetabolitePilot™ Software version 2.0 (SCIEX). The MS/MS spectrum of IVM was exported as text files by the PeakView software (SCIEX) and imported to MetabolitePilot™ software (SCIEX) as a reference spectrum for creating the IVM library. Raw data files (.wiff) of metabolite sample analyses were imported to MetabolitePilot™ software and compared against the IVM‐library peak finding strategies as described in the supplementary material (Supplement Appendix S1). For the LC‐SPE‐NMR/MS system, the HPLC was operated by Hystar 3.2 (Bruker Daltonics), mass spectrum acquired by Microtof control (Bruker Daltonics), and the NMR spectrometer was operated by Topspin 3.5 (Bruker Biospin). The Complete Molecular Confidence – Structure Elucidation (CMC‐se) software version 2.6.1 (Bruker Biospin) was used for structure elucidation.

## 3 RESULTS

### 3.1 *In vitro* and *in vivo* metabolite identification by UHPLC‐Q‐TOF‐MS

**FIGURE 2:**
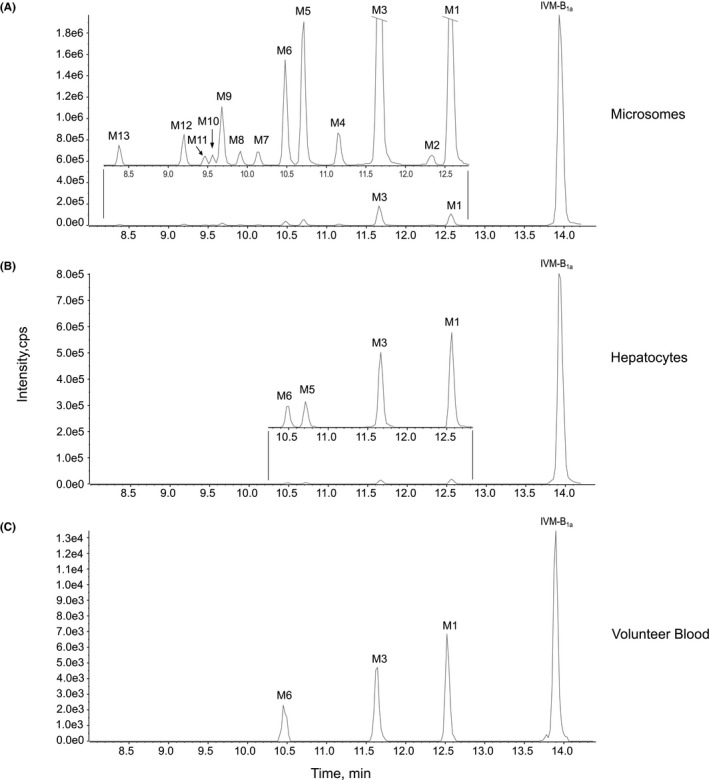
Metabolite chromatograms. Chromatogram (A) shows the metabolites identified using pooled human liver microsomes incubated with IVM for 60 min. The upper insert chromatogram shows a zoom of RT 8.2–12.8 min. Chromatogram (B) shows the metabolites identified in the media fraction of primary human hepatocytes exposed to IVM for 24 hours. The upper insert chromatogram shows a zoom of RT 10.3–12.8 min. Chromatogram (C) shows the metabolites identified in human volunteer blood 24 hours after IVM administration

**FIGURE 3:**
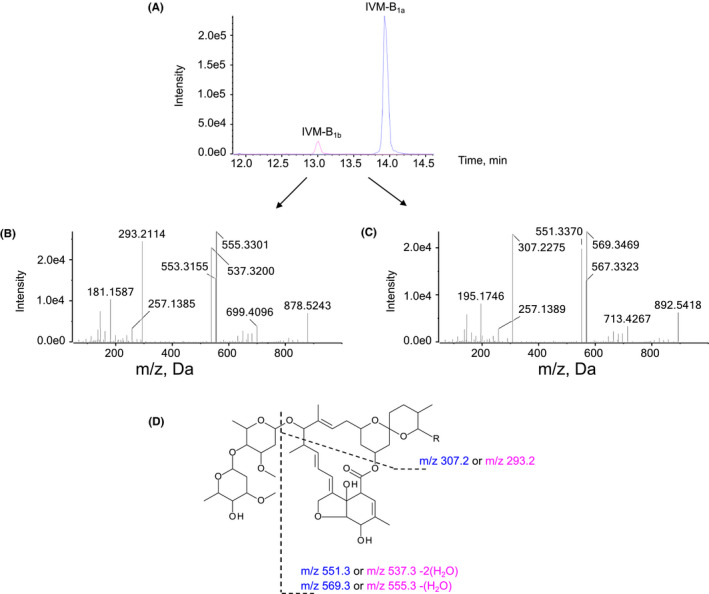
Extracted ion chromatogram and MS/MS spectra. The figure shows (A) extracted ion chromatogram of IVM‐B_1a_ and IVM‐B_1b_, (B) MS/MS spectrum of IVM‐B_1b_, (C) MS/MS spectrum of IVM‐B_1a_, and (D) major fragments of IVM

**FIGURE 4:**
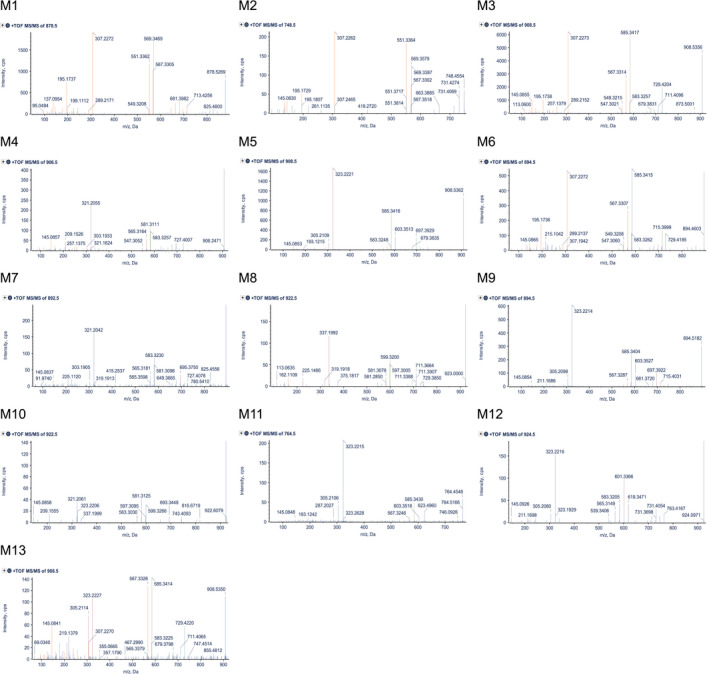
MS/MS spectra of metabolite M1 to M13. Characteristic fragment ion peaks of metabolites matched to ivermectin library peaks are shown in orange. Various characteristic neutral losses ion peaks are shown in different colors

Thirteen IVM metabolites (M1‐M13) were identified from incubations with pooled human liver microsomes as shown in the metabolite chromatogram in Figure 2A. All metabolites were more polar than the IVM parent compound. Four of these metabolites (i.e., M1, M3, M5, and M6) were found also in the medium of IVM‐exposed primary human hepatocytes (Figure 2B). The three most abundant metabolites found in microsome and hepatocytes incubations (i.e., M1, M3, and M6) were found also in healthy volunteer blood 24 hours after IVM administration (Figure 2C). Reference MS/MS spectra of IVM‐B_1a_ and IVM‐B_1b_ used in the library search were generated from analysis of 100 ng/ml of IVM standard solution. IVM‐B_1b_ (*m*/*z* 878.5) eluted 0.9 min earlier than IVM‐B_1a_ (*m*/*z* 892.5) as shown in the extract ion chromatogram (Figure 3A). The major product ions of IVM‐B_1b_ were *m*/*z* 293.2, 537.3, and 555.3 (Figure 3B) and those of IVM‐B_1a_ were *m*/*z* 307.2, 551.3, and 569.3 (Figure 3C). The fragmentation pattern of the ammonium adduct ion of IVM‐B_1a_ and IVM‐B_1b_ is presented in Figure 3D. The MS/MS spectra of M1 to M13 (Figure 4) were used to define the molecular structure of the metabolites.

### 3.2 Metabolite interpretation by LC‐MS/MS

The thirteen metabolites (M1 to M13) identified from the MetabolitePilot™ software (Sciex) are reported in Table 1. The molecular ions that were detected in negative controls (including 0‐min incubations, negative co‐factor controls, and negative IVM controls) were excluded from the list. Percentage scores were based on three parameters including mass accuracy, mass defect,^20^, ^21^ and MS/MS spectrum (for details see supplementary material). Chemical structures and biotransformation sites of the metabolites (M1 to M13) were interpreted based on the two fragment ions of IVM‐B_1a_ (*m*/*z* 307.2 and 551.3; Figure 3). IVM‐B_1a_ metabolites without ion *m*/*z* 307.2 suggested that biotransformation occurred in the spiroketal moiety. The presence of ion *m*/*z* 307.2 without ion *m*/*z* 551.3 indicated that biotransformation occurred in the cyclohexene cyclic ether moiety, and metabolites with altered molecular ions with the presence of both ions *m*/*z* 307.2 and 551.3 indicated that biotransformation occurred in the disaccharide moiety. Identical results were observed for IVM‐B_1b_ metabolites, but with ions *m*/*z* 293.2 and 537.3 (Figure 3). A summary of the interpretations of M1 to M13 is detailed in Table 2. The microsome reactions of pure IVM‐B_1a_ and IVM‐B_1b_ substrates confirmed an identical transformation pattern of both compounds (Table S1). The relative abundance of ivermectin and metabolites, generated using specific cDNA‐expressed human cytochrome P450 enzymes, is provided in the supplementary information (Table S2).

**TABLE 1:** Identification of IVM metabolites from human liver microsomes, primary human hepatocytes, and human volunteer blood

Metabolite ID	Molecular Ion	Formula	Neutral Mass	*m*/*z*	Mass Accuracy (ppm)	R.T. (min)	Peak Area	Peak Area (%)	Score (%)	Experiment
IVM‐B_1a_	Parent‐B_1a_ [M+NH_4_]^+^	C_48_H_74_O_14_	874.5083	892.5421	0.5	13.94	1.03E+07	—	93.7	HLM
M1	Demethylation [M+NH_4_]^+^	C_47_H_72_O_14_	860.4922	878.5260	0.0	12.58	6.07E+05	26.38	86.6	HLM, PHH, Blood
M2	Loss of C_7_H_12_O_3_ [M+NH_4_]^+^	C_41_H_62_O_11_	730.4283	748.4621	−1.2	12.33	2.76E+04	1.20	81.7	HLM
M3	Oxidation [M+NH_4_]^+^	C_48_H_74_O_15_	890.5033	908.5371	0.6	11.67	9.03E+05	39.24	78.9	HLM, PHH, Blood
M4	Ketone formation [M+NH_4_]^+^	C_48_H_72_O_15_	888.4862	906.5201	−1.0	11.17	6.42E+04	2.79	69.7	HLM
M5	Oxidation [M+NH_4_]^+^	C_48_H_74_O_15_	890.5030	908.5368	0.2	10.72	2.96E+05	12.86	68.7	HLM, PHH
M6	Demethylation and oxidation [M+NH_4_]^+^	C_47_H_72_O_15_	876.4868	894.5206	−0.4	10.48	1.63E+05	7.08	76.5	HLM, PHH, Blood
M7	Demethylation and ketone formation [M+NH_4_]^+^	C_47_H_70_O_15_	874.4708	892.5046	−0.8	10.15	2.20E+04	0.96	67.0	HLM
M8	Demethylation to carboxylic acid [M+NH_4_]^+^	C_48_H_72_O_16_	904.4808	922.5146	−1.3	9.92	1.81E+04	0.79	66.7	HLM
M9	Demethylation and oxidation [M+NH_4_]^+^	C_47_H_72_O_15_	876.4870	894.5208	−0.2	9.69	9.56E+04	4.15	71.7	HLM
M10	Demethylation to carboxylic acid [M+NH_4_]^+^	C_48_H_72_O_16_	904.4819	922.5157	−0.1	9.57	1.34E+04	0.58	66.7	HLM
M11	Loss of C_7_H_12_O_3_ and oxidation [M+NH_4_]^+^	C_41_H_62_O_12_	746.4225	764.4563	−2.2	9.48	1.36E+04	0.59	67.3	HLM
M12	Dioxidation [M+NH_4_]^+^	C_48_H_74_O_16_	906.4976	924.5314	−0.1	9.22	4.89E+04	2.13	68.4	HLM
M13	Oxidation [M+NH_4_]^+^	C_48_H_74_O_15_	890.5027	908.5365	−0.1	8.40	2.86E+04	1.24	73.7	HLM

Relative peak area was calculated as individual metabolite peak area divided on total metabolite peak area.

Abbreviations: Blood, human volunteer blood; HLM, human liver microsomes; *m*/*z*, mass‐to‐charge ratio; PHH, primary human hepatocytes; ppm, parts per million; R.T., retention time.

**TABLE 2:** Molecular structures of metabolites predicted by accurate mass and mass fragmentation patterns

Metabolite derivative	Biotransformation	Metabolite	Molecular ion (*m*/*z*)	Structure elucidation
Hydroxylate	Demethylation (R‐CH_3_ to R‐H)	M1	878.5	Presence of ion *m*/*z* 307.2 indicates no biotransformation on spiroketal of IVM‐B_1a_
				Presence of ion *m*/*z* 551.3 indicates no biotransformation on cyclohexene cyclic ether of IVM‐B_1a_
				Molecular ion reduced 14 Da relative to IVM‐B_1a_ indicates demethylation occurred on the disaccharide (892.5–14 Da)
	Oxidation (+O)	M3	908.5	Presence of ion *m*/*z* 307.2 indicates no biotransformation on the spiroketal of IVM‐B_1a_
				Absence of ion *m*/*z* 551.3 and presence of *m*/*z* 567.3 indicate oxidation on the cyclohexene cyclic ether of IVM‐B_1a_ (551.3 + 16 Da)
		M5	908.5	Absence of ion *m*/*z* 307.2 and presence of *m*/*z* 323.2 indicate oxidation on the spiroketal of IVM‐B_1a_ (307.2 + 16 Da)
		M13	908.5	Absence of ion *m*/*z* 307.2 and presence of *m*/*z* 323.2 indicate oxidation on the spiroketal of IVM‐B_1a_ (307.2 + 16 Da)
	Demethylation (R‐CH_3_ to R‐H) and oxidation (+O)	M6	894.5	Presence of ion *m*/*z* 307.2 indicates no biotransformation on the spiroketal of IVM‐B_1a_
				Absence of ion *m*/*z* 551.3 and presence of *m*/*z* 567.3 indicate oxidation on the cyclohexene cyclic ether of IVM‐B_1a_ (551.3 + 16 Da)
				Molecular ion increased 2 Da relative to IVM‐B_1a_ indicates the combination of oxidation (+16 Da) and demethylation (−14 Da), where demethylation occur on the disaccharide
		M9	894.5	Absence of ion *m*/*z* 307.2 and presence of *m*/*z* 323.2 indicate oxidation on the spiroketal of IVM‐B_1a_ (307.2 + 16 Da)
				Molecular ion increased 2 Da relative to IVM‐B_1a_ indicates the combination of oxidation (+16 Da) and demethylation (−14 Da), where demethylation occur on the disaccharide
	Dioxidation (+2O)	M12	924.5	Absence of ion *m*/*z* 307.2 and presence of *m*/*z* 323.2 indicate oxidation on the spiroketal of IVM‐B_1a_ (307.2 + 16 Da)
				Absence of ion *m*/*z* 551.3 and presence of *m*/*z* 583.3 indicate two oxidations on the cyclohexene cyclic ether of IVM‐B_1a_ (551.3 + 32 Da)
Monosaccharide	Loss of monosaccharide	M2	748.5	Presence of ion *m*/*z* 307.2 indicates no biotransformation on the spiroketal of IVM‐B_1a_
				Presence of ion *m*/*z* 551.3 indicates no biotransformation on the cyclohexene cyclic ether of IVM‐B_1a_
				Molecular ion reduced 144 Da relative to IVM‐B_1a_ indicates loss of the monosaccharide moiety (892.4 −144 Da)
		M11	764.5	Absence of ion *m*/*z* 307.2 and presence of *m*/*z* 323.2 indicate oxidation on the spiroketal of M2 (748.5 + 16 Da)
Ketone	Ketone formation (R‐CH_2_‐R’ to R‐CO‐R’)	M4	906.5	Absence of ion *m*/*z* 307.2 and presence of *m*/*z* 321.2 indicate ketone formation on the spiroketal of IVM‐B_1a_ (307.2 + 14 Da)
		M7	892.5	Absence of ion *m*/*z* 307.2 and presence of *m*/*z* 321.2 indicate ketone formation on the spiroketal of IVM‐B_1a_ (307.2 + 14 Da)
				Molecular ion not changed relative to IVM‐B_1a_ indicates demethylation on the disaccharide (892.5 + 14 Da–14 Da)
Carboxylate	Oxidation and ketone formation (+O‐CH_2_+CO)	M10	922.5	Absence of ion *m*/*z* 307.2 and presence of *m*/*z* 321.2 indicate ketone formation on the spiroketal of IVM‐B_1a_ (307.2 + 14 Da)
				Absence of ion *m*/*z* 551.3 and presence of *m*/*z* 581.3 indicate oxidations on the cyclohexene cyclic ether of IVM‐B_1a_ (551.3 + 14 Da +16 Da)
		M8	922.5	Absence of ion *m*/*z* 307.2 and presence of *m*/*z* 337.2 indicate carboxy formation on the spiroketal of IVM‐B_1a_ (307.2 + 30 Da)

### 3.3 Confirmation of *in vivo* metabolite structures (M1 and M3) by LC‐SPE‐NMR/MS

**FIGURE 5:**
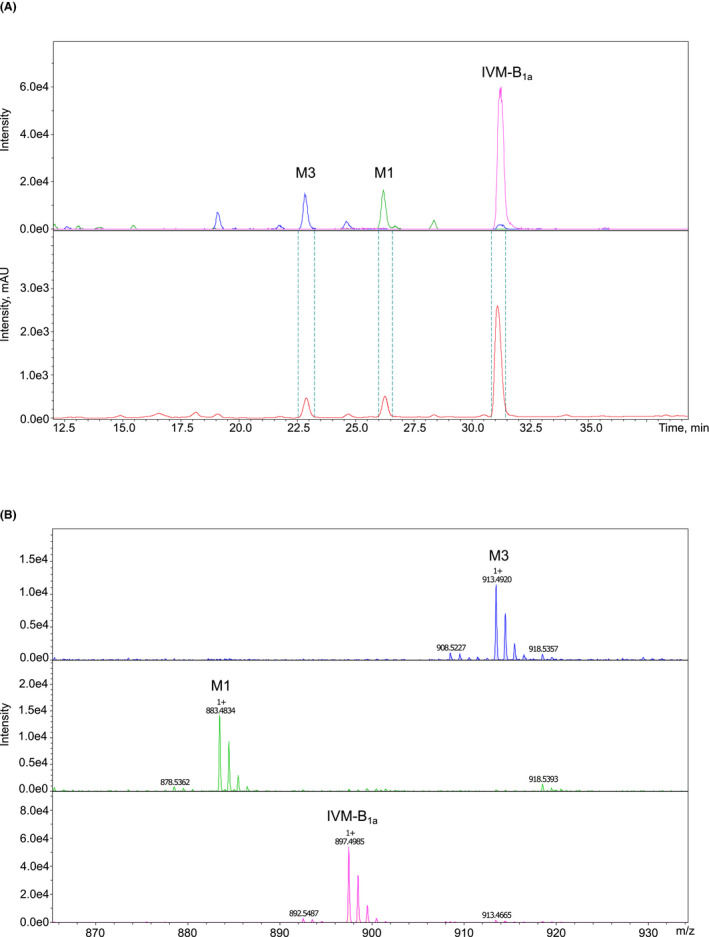
Metabolite purification and detection for NMR analysis. Panel (A) is an extracted ion chromatogram of IVM, M1, and M3 during the preparation chromatography, and UV chromatogram at 240 nm for the trapping experiment (the dotted lines indicate the start and end of the trapping procedure, (B) is a mass spectrum from the peaks in the extracted ion chromatogram demonstrating that the molecules form stable sodium adducts

**FIGURE 6:**
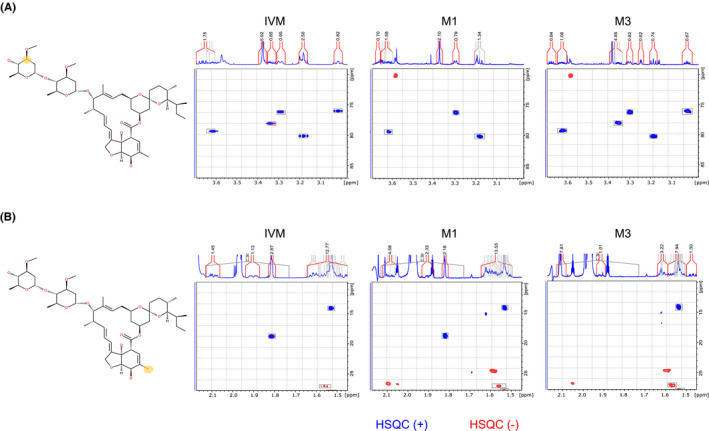
NMR spectra of ivermectin (left) compared with M1 (middle) and M3 (right). The structure of IVM was labeled in yellow at signals showing the most significant differences in the spectra of the metabolites. The cursor in HSQC spectrum indicates signals missing due to changes in the metabolites. Panel (A) the shift of the –CH group in the metabolite M1 (*m*/*z* 883) at the position labeled in yellow and (B) the missing –CH_3_ group of the metabolite M3 (*m*/*z* 913) at position labeled in yellow. The changes in chemical shift values are listed in Table 3

Microsome metabolites were separated by HPLC followed by MS and UV detection (Figure 5A) with subsequent postcolumn trapping of analytes on the SPE cartridges. Molecular formulas of the metabolites were obtained from high‐resolution mass spectrometry during a preparation run. The final isolation of the metabolites was based on UV response in order to reduce the risk of contamination of the ion source because of the large amount of sample injected on the column. M1 and M3 were separated by HPLC and detected by MS as sodium adducts [M+Na]^+^ ions (i.e., *m*/*z* 883 and *m*/*z* 913, respectively) (Figure 5B). The NMR spectra confirmed that the demethylation of M1 occurred at C3″ (labeled in yellow, Figure 6A) and that the hydroxylation of M3 occurred at C4 (labeled in yellow, Figure 6B). The evaluation of HSQC and HMBC spectra of IVM, M1, and M3 shows the location of biotransformation (Table 3, Figure 6, Supplement Appendix S2). Chemical shift values for proton and carbon resonances are shown in Table 3.

**TABLE 3:** Chemical shift values for proton and carbon resonances obtained from HSQC and HMBC spectra of ivermectin, M1 and M3

Position	Ivermectin		M1		M3	
	H‐shift [ppm]	C‐shift [ppm]	H‐shift [ppm]	C‐shift [ppm]	H‐shift [ppm]	C‐shift [ppm]
1	—	171.91	—	171.98	—	171.72
2	3.16	45.61	3.19	45.67	3.25	45.53
3	5.42	118.65	5.42	118.60	5.70	118.64
4	—	136.50	—	136.69	—	**139.78**
5	4.19	67.47	4.20	67.43	**4.41**	**65.22**
6	3.78	80.37	3.79	80.31	3.81	80.48
7	—	80.48	—	80.47	—	80.79
8	—	140.36	—	140.37	—	140.28
9	5.84	120.29	5.84	120.33	5.86	120.55
10	5.89	125.16	5.89	125.15	5.90	125.18
11	5.81	137.39	5.80	137.56	5.81	137.68
12	2.63	39.44	2.63	39.52	2.64	39.55
13	3.96	81.74	3.96	81.72	3.96	39.55
14	—	134.84	—	134.81	—	134.85
15	5.21	118.89	5.21	118.98	5.22	118.94
16 (a/b)	2.31/2.24	33.74	2.31/2.25	33.56	2.32/2.25	33.68
17	3.73	67.42	3.74	67.40	3.74	67.35
18 (a/b)	1.9/0.79	36.50	1.91/0.80	36.51	1.91/0.80	36.55
19	5.04	68.55	5.07	68.44	5.09	68.60
20 (a/b)	2.09/1.25	41.46	2.09/1.26	41.40	2.10/1.27	41.42
21	—	97.43	—	97.41	—	97.46
22 (a/b)	1.61/1.52	35.50	1.60/1.52	35.66	1.61/1.52	35.72
23 (a/b)	1.53/1.52	27.99	1.53/1.52	27.90	1.53/1.52	27.99
24	1.54	31.10	1.54	31.07	1.54	31.09
25	3.29	76.19	3.30	76.28	3.30	76.23
26 (a/b)	4.60/4.61	67.54	4.61/4.62	67.62	4.63/4.62	67.66
CH_3_/CH_2_ (a/b) (4)	1.81	18.79	1.82	18.75	**4.16/4.15**	**62.53**
CH_3_ (12)	1.16	19.68	1.16	19.62	1.17	19.63
CH_3_ (14)	1.53	14.13	1.53	14.04	1.54	14.12
CH_3_ (24)	0.81	16.72	0.82	16.63	0.82	16.72
27	1.62	35.22	1.62	35.17	1.63	35.22
CH_3_ (27)	0.97	11.74	0.89	11.62	0.89	11.63
28 (a/b)	1.56/1.40	27.03	1.56/1.41	27.03	1.57/1.41	27.01
CH_3_ (28)	0.89	11.69	0.97	11.65	0.98	11.67
1′	4.78	94.89	4.79	94.81	4.79	94.90
2′ (a/b)	2.30/1.51	34.31	2.30/1.53	34.07	2.29/1.53	34.22
3′	3.62	79.34	3.62	79.45	3.62	79.30
4′	3.18	80.13	3.18	80.15	3.19	80.25
5′	3.83	67.14	3.83	67.05	3.84	67.15
CH_3_ (3′)	3.37	55.74	3.37	55.69	3.38	55.57
CH_3_ (5′)	1.23	17.95	1.24	17.92	1.24	17.98
1″	5.34	98.04	5.31	97.95	5.34	98.08
2″ (a/b)	2.26/1.46	34.55	**2.07/1.59**	**38.02**	2.27/1.47	34.69
3″	3.33	78.05	**3.64**	**68.43**	3.35	78.06
4″	3.01	76.01	**2.92**	**77.70**	3.02	76.14
5″	3.66	68.42	3.67	68.44	3.68	68.45
CH_3_ (3″)	3.38	56.09	—	—	3.38	56.27
CH_3_ (5″)	1.20	17.11	1.20	17.01	1.20	17.08

For labeling of positions refer to Figure 1. Bold fonts indicate places in the molecule where changes have occured.

As the sensitivity of the 500 MHz instrument was not sufficient to obtain an HMBC spectrum within the period of the nitrogen refill cycle (one week), the isolated metabolites were analyzed using an 800 MHz instrument. This provided a complete set of NMR data (within 72 h) for structure elucidation. The HMBC correlations for IVM, M1, and M3 together with the proton spectrum obtained at 800 MHz are detailed in the NMR supplement report.

### 3.4 Metabolic pathway characterized by pure human CYP enzyme

**FIGURE 7:**
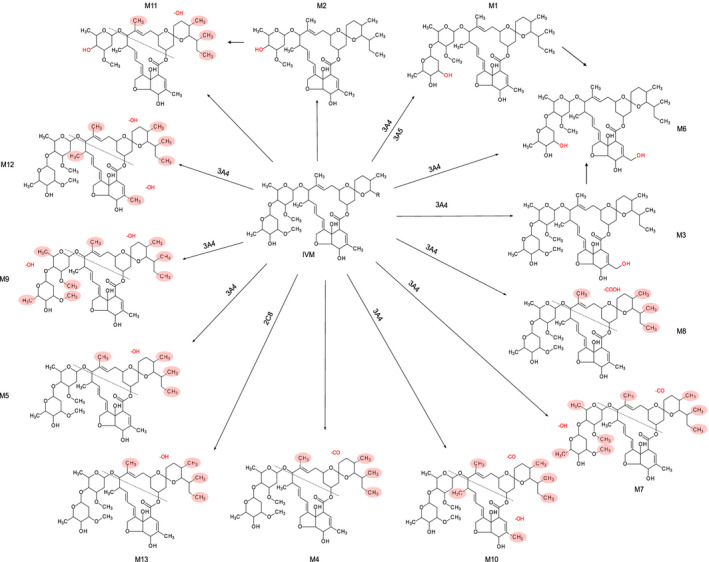
Proposed metabolic pathway of ivermectin metabolites. The exact site of biotransformation could not be confirmed for some metabolites. In these, the potential biotransformation sites are highlighted in red shading

IVM metabolites M1, M3, M5, M6, M7, M8, M9, M10, and M12 were all found after 60 min of incubation with CYP3A4. IVM metabolite M1 was also produced by CYP3A5. M13 was produced by CYP2C8. None of the other investigated enzymes produced detectable amounts of the identified IVM metabolites. Proposed metabolite pathways are shown in Figure 7.

## 4 DISCUSSION

The peak area of metabolites, relative to IVM, was used to estimate the relative abundance of each metabolite. In 60‐min microsomes reactions, the five most abundant metabolites were M3 > M1 > M5 > M6 > M9 (Table 1, Figure 2). The four most abundant of these metabolites were also present in IVM‐exposed primary human hepatocytes (M1 > M3 > M5 > M6). Three of these metabolites were found also in human volunteer blood samples taken 24 h after IVM administration (M1 > M3 > M6). This is the first report of IVM metabolites identified from human hepatocytes and clinical blood samples.

The IVM demethylation, oxidation, and monosaccharide metabolites identified from microsomes in this study were consistent with those reported previously from human microsomes.^18^ However, four additional IVM metabolites, including ketone and carboxylic derivatives, were also found in our study. Advancements in UHPLC technology^22^ and state‐of‐the‐art high‐resolution mass spectrometry^23^, ^24^, ^25^ used here improved the sensitivity for metabolite detection. By using pure CYP enzymes, we showed that IVM is primarily metabolized by CYP3A4. However, we identified two additional CYP metabolism pathways of IVM; the demethylated IVM metabolite (M1) was also produced by CYP3A5 and an additional hydroxylated IVM metabolite (M13) was produced by CYP2C8. Metabolite 13 might be considered a minor metabolism pathway since it contributed <1.5% to total metabolite peak area when ivermectin was incubated for 60 min with pooled human liver microsomes. However, it is possible that this metabolism pathway could be relatively more important in patients with functional polymorphisms in the CYP3A4 and/or CYP3A5 genes, resulting in reduced activity of CYP3A4/5 enzymes. All major metabolites identified by microsome and hepatocyte incubations, and in clinical samples were produced by CYP3A4. Metabolite 1, which was the most abundant metabolite in hepatocyte incubations and in clinical samples, was produced both by CYP3A4 and CYP3A5. However, data presented here cannot be used to elucidate the relative *in vivo* contribution of each of these isoenzymes. Thus, it is likely that functional polymorphisms in CYP3A4 and/or CYP3A5 genes could be of clinical importance, since it might affect the pharmacokinetic properties of ivermectin and its metabolites. Indeed, polymorphisms in the multidrug resistance (MDR1) gene, coding for p‐glycoprotein, and in the CYP3A gene have been linked to altered treatment response of IVM when used in onchocerciasis in a Ghanaian population.^26^ This warrants further pharmacogenomic evaluations in larger patient populations to elucidate the risk of treatment failures and/or adverse events in patients with functional polymorphisms.

The exact chemical structures of the two most abundant *in vivo* metabolites (M1 and M3) were obtained by NMR, but we could not generate enough material for NMR evaluation of the third *in vivo* metabolite (M6). The metabolic pathway data suggest that M1, M3, and M6 were all produced by CYP3A4, and that M6 is a combination of the demethylation and oxidation seen in M1 and M3, respectively. Thus, we propose that M6 is 3″‐*O*‐demethyl, 4‐hydroxymethyl‐ivermectin, a further (common) metabolite product of M1 and M3. With two sites of transformation occurring in M6 (demethylation and oxidation), it is more polar and elutes earlier than M1 and M3. Additional reversed‐phase chromatography data support the M6 structure based on the elution order. The elution order in this study is also consistent with the study of Zeng et al..^18^

Interestingly, many low abundance metabolites produced in microsomes were not detected from primary human hepatocytes in culture or from human volunteer blood after IVM administration. Several factors could influence the metabolic function of hepatocytes *in vitro*, such as initial cell suspension, confluence density of adherent cells, and drug concentration. The lower number of metabolites found in volunteer blood samples compared to microsomes could be because of phospholipids in blood samples. *In vitro* systems are also more efficient in producing metabolites and do not have elimination pathways (such as renal elimination) compared to *in vivo* systems, which could have an impact on detection results. We suggest future studies to characterize IVM metabolites produced at later time points from both hepatocytes and human blood, when metabolite abundance may possibly be altered compared to the 24‐h time point thus allowing characterization of *in vivo* metabolism over time. To support these efforts, we have performed a clinical trial (NCT03690453) to assess the pharmacokinetic profiles of key IVM metabolites in orally treated volunteers over several weeks, and evaluated their potential mosquito‐lethal and antimalarial effects. A better understanding of IVM metabolite pharmacokinetics can provide further insight into pharmacodynamics and efficacy for NTDs, especially those requiring multiple administrations such as scabies and strongyloidiasis. Furthermore, these IVM metabolites may inhibit viral replication and should be evaluated against SARS‐CoV‐2, the causative agent of COVID‐19.

## 5 CONCLUSION

We report for the first time, novel IVM metabolites from human liver microsomes, primary human hepatocytes, and from human blood after oral IVM dosing. Importantly, we identified and confirmed the structure of the two major IVM metabolites in humans; 3″‐*O*‐demethylation IVM (M1) and 4‐hydroxymethyl IVM (M3).

## CONFLICT OF INTEREST

The authors declare no conflict of interest. We did not purchase any of the compounds or instruments mentioned in this article from Bruker.

## AUTHOR’S CONTRIBUTIONS

P.T., K.C.K., and J.T. designed the research. P.T. performed MS experiments. P.T. analyzed data and wrote the first draft of the manuscript. M.G. performed NMR experiments. P.J. and B.H. performed the clinical trial. A.R. and J.H.A. performed the hepatocytes experiments. All the authors contributed to the organization of the study and the final draft of the manuscript.

## ETHICAL STATEMENT

Venous blood was collected from three healthy Thai volunteers given a single dose of IVM (400 µg/kg). The trial was conducted at the Hospital of Tropical Medicine, Faculty of Tropical Medicine, Mahidol University in Bangkok, Thailand. The study protocol was approved by the ethics committees of the Faculty of Tropical Medicine, Mahidol University (reference number TMEC 15–004, approval number MUTM 2015–016–02), by the Oxford University Tropical Research Ethics Committee (OXTREC 4–15), and the Walter Reed Army Institute of Research (WRAIR#2228). The trial was registered at ClinicalTrials.gov number NCT02568098. Each volunteer was provided with an explanation of the study and signed a written informed consent before study entry.

## Supporting information

Table S1

Table S2

Appendix S1

Appendix S2

## Data Availability

The data that support the findings of this study are available from the corresponding author upon reasonable request.

## References

[1] Gonzalez P , Gonzalez FA , Ueno K . Ivermectin in human medicine, an overview of the current status of its clinical applications. Curr Pharm Biotechnol. 2012;13(6):1103‐1109. 22039800 10.2174/138920112800399248

[2] Caly L , Druce JD , Catton MG , Jans DA , Wagstaff KM . The FDA‐approved Drug Ivermectin inhibits the replication of SARS‐CoV‐2 in vitro. Antiviral Res. 2020;178:104787. 32251768 10.1016/j.antiviral.2020.104787 PMC7129059

[3] Chaccour CJ , Kobylinski KC , Bassat Q , et al. Ivermectin to reduce malaria transmission: a research agenda for a promising new tool for elimination. Malar J. 2013;12:153. 23647969 10.1186/1475-2875-12-153 PMC3658945

[4] Smit MR , Ochomo EO , Aljayyoussi G , et al. Safety and mosquitocidal efficacy of high‐dose ivermectin when co‐administered with dihydroartemisinin‐piperaquine in Kenyan adults with uncomplicated malaria (IVERMAL): a randomised, double‐blind, placebo‐controlled trial. Lancet Infect Dis. 2018;18(6):615‐626. 29602751 10.1016/S1473-3099(18)30163-4

[5] Kobylinski KC , Jittamala P , Hanboonkunupakarn B , et al. Safety, pharmacokinetics, and mosquito‐lethal effects of ivermectin in combination with dihydroartemisinin‐piperaquine and primaquine in healthy adult Thai subjects. Clin Pharmacol Ther. 2020;107(5):1221‐1230. 31697848 10.1002/cpt.1716 PMC7285759

[6] Kobylinski KC , Foy BD , Richardson JH . Ivermectin inhibits the sporogony of Plasmodium falciparum in Anopheles gambiae. Malar J. 2012;11:381. 23171202 10.1186/1475-2875-11-381 PMC3519548

[7] Pinilla YT , Lopes SCP , Sampaio VS , et al. Promising approach to reducing Malaria transmission by ivermectin: Sporontocidal effect against Plasmodium vivax in the South American vectors Anopheles aquasalis and Anopheles darlingi. PLoS Negl Trop Dis. 2018;12(2):e0006221. 29444080 10.1371/journal.pntd.0006221 PMC5828505

[8] Kobylinski KC , Ubalee R , Ponlawat A , et al. Ivermectin susceptibility and sporontocidal effect in Greater Mekong Subregion Anopheles. Malar J. 2017;16(1):280. 28687086 10.1186/s12936-017-1923-8 PMC5501099

[9] Alout H , Krajacich BJ , Meyers JI , et al. Evaluation of ivermectin mass drug administration for malaria transmission control across different West African environments. Malar J. 2014;13:417. 25363349 10.1186/1475-2875-13-417 PMC4226880

[10] Foy BD , Alout H , Seaman JA , et al. Efficacy and risk of harms of repeat ivermectin mass drug administrations for control of malaria (RIMDAMAL): a cluster‐randomised trial. Lancet. 2019;393(10180):1517‐1526. 30878222 10.1016/S0140-6736(18)32321-3 PMC6459982

[11] Campbell WC . Ivermectin and Abamectin, 1st edn New York: Springer‐Verlag; 1989.

[12] Campbell WC . Ivermectin: an update. Parasitol Today. 1985;1(1):10‐16. 15275618 10.1016/0169-4758(85)90100-0

[13] Miwa GT , Walsh JS , VandenHeuvel WJ , et al. The metabolism of avermectins B1a, H2B1a, and H2B1b by liver microsomes. Drug Metab Dispos. 1982;10(3):268‐274. 6125361

[14] Chiu SH , Sestokas E , Taub R , Smith JL , Arison B , Lu AY . The metabolism of avermectin‐H2B1a and ‐H2B1b by pig liver microsomes. Drug Metab Dispos. 1984;12(4):464‐469. 6148214

[15] Chiu SH , Sestokas E , Taub R , et al. Metabolic disposition of ivermectin in tissues of cattle, sheep, and rats. Drug Metab Dispos. 1986;14(5):590‐600. 2876867

[16] Chiu SH , Carlin JR , Taub R , et al. Comparative metabolic disposition of ivermectin in fat tissues of cattle, sheep, and rats. Drug Metab Dispos. 1988;16(5):728‐736. 2906598

[17] Chiu SH , Taub R , Sestokas E , Lu AY , Jacob TA . Comparative in vivo and in vitro metabolism of ivermectin in steers, sheep, swine, and rat. Drug Metab Rev. 1987;18(2–3):289‐302. 3330518 10.3109/03602538708998309

[18] Zeng Z , Andrew NW , Arison BH , Luffer‐Atlas D , Wang RW . Identification of cytochrome P4503A4 as the major enzyme responsible for the metabolism of ivermectin by human liver microsomes. Xenobiotica. 1998;28(3):313‐321. 9574819 10.1080/004982598239597

[19] Roth A , Maher SP , Conway AJ , et al. A comprehensive model for assessment of liver stage therapies targeting Plasmodium vivax and Plasmodium falciparum. Nat Commun. 2018;9(1):1837. 29743474 10.1038/s41467-018-04221-9 PMC5943321

[20] Sleno L . The use of mass defect in modern mass spectrometry. J Mass Spectrom. 2012;47(2):226‐236. 22359333 10.1002/jms.2953

[21] Zhang H , Zhang D , Ray K , Zhu M . Mass defect filter technique and its applications to drug metabolite identification by high‐resolution mass spectrometry. J Mass Spectrom. 2009;44(7):999‐1016. 19598168 10.1002/jms.1610

[22] Churchwell MI , Twaddle NC , Meeker LR , Doerge DR . Improving LC‐MS sensitivity through increases in chromatographic performance: comparisons of UPLC‐ES/MS/MS to HPLC‐ES/MS/MS. J Chromatogr B Analyt Technol Biomed Life Sci. 2005;825(2):134‐143. 10.1016/j.jchromb.2005.05.037 16002352

[23] Theodoridis GA , Gika HG , Want EJ , Wilson ID . Liquid chromatography‐mass spectrometry based global metabolite profiling: a review. Anal Chim Acta. 2012;711:7‐16. 22152789 10.1016/j.aca.2011.09.042

[24] Meyer MR , Maurer HH . Current applications of high‐resolution mass spectrometry in drug metabolism studies. Anal Bioanal Chem. 2012;403(5):1221‐1231. 22349341 10.1007/s00216-012-5807-z

[25] Ramanathan R , Jemal M , Ramagiri S , et al. It is time for a paradigm shift in drug discovery bioanalysis: from SRM to HRMS. J Mass Spectrom. 2011;46(6):595‐601. 21630388 10.1002/jms.1921

[26] Kudzi W , Dodoo AN , Mills JJ . Genetic polymorphisms in MDR1, CYP3A4 and CYP3A5 genes in a Ghanaian population: a plausible explanation for altered metabolism of ivermectin in humans? BMC Med Genet. 2010;11:111. 20630055 10.1186/1471-2350-11-111 PMC3161347

