# Supplementary material for: Identification of the metabolites of ivermectin in humans
Source: Pharmacol Res Perspect. 2021 Jan 26;9(1):e00712. doi: 10.1002/prp2.712 (PMC7836931; doi:10.1002/prp2.712)
Supplement: Supplementary file 1 — Table S1 [file PRP2-9-e00712-s001.pdf]

Table S1. Identification of IVM-B_{1a} and IVM-B_{1b} metabolites in human liver microsomes.

Metabolite ID	Molecular Ion	Formula	Neutral Mass	m/z	Mass Accuracy (ppm)	R.T. (min)	% Score
IVM-B_{1a}	Parent B_{1a} $[M+NH_4]^+$	$C_{48}H_{74}O_{14}$	874.51	892.5421	0.5	13.95	92.7
IVM-B_{1b}	Parent B_{1b} $[M+NH_4]^+$	$C_{47}H_{72}O_{14}$	860.49	878.5257	-0.4	13.05	93.9
M1-B_{1a}	Demethylation $[M+NH_4]^+$	$C_{47}H_{72}O_{14}$	860.49	878.5263	0.3	12.58	89.1
M1-B_{1b}	Demethylation $[M+NH_4]^+$	$C_{46}H_{70}O_{14}$	846.48	864.5100	-0.4	11.77	87.3
M2-B_{1a}	Loss of $C_7H_{12}O_3$ $[M+NH_4]^+$	$C_{41}H_{62}O_{11}$	730.43	748.4623	-0.9	12.35	80.8
M2-B_{1b}	Loss of $C_7H_{12}O_3$ $[M+NH_4]^+$	$C_{40}H_{60}O_{11}$	716.41	734.4468	-0.8	11.56	78.9
M3-B_{1a}	Oxidation $[M+NH_4]^+$	$C_{48}H_{74}O_{15}$	890.50	908.5368	0.2	11.67	78.9
M3-B_{1b}	Oxidation $[M+NH_4]^+$	$C_{47}H_{72}O_{15}$	876.49	894.5205	-0.5	10.85	81.8
M4-B_{1a}	Ketone formation $[M+NH_4]^+$	$C_{48}H_{72}O_{15}$	888.49	906.5206	-0.3	11.17	69.9
M4-B_{1b}	Ketone formation $[M+NH_4]^+$	$C_{47}H_{70}O_{15}$	874.47	892.5050	-0.3	10.46	68.9
M5-B_{1a}	Oxidation $[M+NH_4]^+$	$C_{48}H_{74}O_{15}$	890.50	908.5370	0.5	10.71	70.8
M5-B_{1b}	Oxidation $[M+NH_4]^+$	$C_{47}H_{72}O_{15}$	876.49	894.5204	-0.6	9.83	71.1
M6-B_{1a}	Demethylation and oxidation $[M+NH_4]^+$	$C_{47}H_{72}O_{15}$	876.49	894.5209	-0.1	10.49	76.7
M6-B_{1b}	Demethylation and oxidation $[M+NH_4]^+$	$C_{46}H_{70}O_{15}$	862.47	880.5047	-0.7	9.79	77.1
M7-B_{1a}	Demethylation and ketone formation $[M+NH_4]^+$	$C_{47}H_{70}O_{15}$	874.47	892.5057	0.5	10.14	67.4
M8-B_{1a}	Demethylation to carboxylic acid $[M+NH_4]^+$	$C_{48}H_{72}O_{16}$	904.48	922.5155	-0.4	9.92	67.5
M9-B_{1a}	Demethylation and oxidation $[M+NH_4]^+$	$C_{47}H_{72}O_{15}$	876.49	894.5204	-0.7	9.69	70.9
M9-B_{1b}	Demethylation and oxidation $[M+NH_4]^+$	$C_{46}H_{70}O_{15}$	862.47	880.5048	-0.5	8.94	66.7
M10-B_{1a}	Demethylation to carboxylic acid $[M+NH_4]^+$	$C_{48}H_{72}O_{16}$	904.48	922.5161	0.2	9.57	66.7
M11-B_{1a}	Loss of $C_7H_{12}O_3$ and oxidation $[M+NH_4]^+$	$C_{41}H62O_{12}$	746.42	764.4568	-1.6	9.45	68.9
M12-B_{1a}	Dioxidation $[M+NH_4]^+$	$C_{48}H_{74}O_{16}$	906.50	924.5306	-1.0	9.20	68.0
M12-B_{1b}	Dioxidation $[M+NH_4]^+$	$C_{47}H_{72}O_{16}$	892.48	910.5149	-1.1	8.44	66.7
M13-B_{1a}	Oxidation $[M+NH_4]^+$	$C_{48}H_{74}O_{15}$	890.50	908.5361	-0.5	8.37	73.6
M13-B_{1b}	Oxidation $[M+NH_4]^+$	$C_{47}H_{72}O_{15}$	876.49	894.5205	-0.5	7.91	74.2

m/z = mass-to-charge ratio, ppm = parts per million, R.T. = retention time.
