# Supplementary material for: Identification of the metabolites of ivermectin in humans
Source: Pharmacol Res Perspect. 2021 Jan 26;9(1):e00712. doi: 10.1002/prp2.712 (PMC7836931; doi:10.1002/prp2.712)
Supplement: Supplementary file 2 — Table S2 [file PRP2-9-e00712-s002.pdf]

Table S2. Relative peak area of ivermectin and metabolites generated in specific rCYP reactions.

rCYP	Metabolite ID	Molecular Ion	Formula	Neutral Mass	m/z	Mass Accuracy (ppm)	R.T. (min)	Peak area (%)
3A4	IVM-B_{1a}	Parent-B_{1a} $[M+NH_4]^+$	$C_{48}H_{74}O_{14}$	874.5070	892.5408	-1.0	14.33	9.1
	M1	Demethylation $[M+NH_4]^+$	$C_{47}H_{72}O_{14}$	860.4902	878.5240	-2.3	12.85	11.1
	M3	Oxidation $[M+NH_4]^+$	$C_{48}H_{74}O_{15}$	890.5015	908.5354	-1.3	11.89	3.6
	M5	Oxidation $[M+NH_4]^+$	$C_{48}H_{74}O_{15}$	890.5022	908.5360	-0.7	10.90	0.5
	M6	Demethylation and oxidation $[M+NH_4]^+$	$C_{47}H_{72}O_{15}$	876.4864	894.5203	-0.8	10.67	14.8
	M7	Demethylation and ketone formation $[M+NH_4]^+$	$C_{47}H_{70}O_{15}$	874.4697	892.5035	-2.0	10.31	1.9
	M8	Demethylation to carboxylic acid $[M+NH_4]^+$	$C_{48}H_{72}O_{16}$	904.4816	922.5154	-0.5	10.09	4.3
	M9	Demethylation and oxidation $[M+NH_4]^+$	$C_{47}H_{72}O_{15}$	876.4862	894.5201	-1.0	9.83	2.7
	M10	Demethylation to carboxylic acid $[M+NH_4]^+$	$C_{48}H_{72}O_{16}$	904.4828	922.5167	0.9	9.73	0.6
	M12	Dioxidation $[M+NH_4]^+$	$C_{48}H_{74}O_{16}$	906.4969	924.5308	-0.8	9.33	0.9
3A5	IVM-B_{1a}	Parent-B_{1a} $[M+NH_4]^+$	$C_{48}H_{74}O_{14}$	874.5080	892.5418	0.1	14.35	65.4
	M1	Demethylation $[M+NH_4]^+$	$C_{47}H_{72}O_{14}$	860.4926	878.5264	0.4	12.84	4.0
2C8	IVM-B_{1a}	Parent-B_{1a} $[M+NH_4]^+$	$C_{48}H_{74}O_{14}$	874.5080	892.5418	0.1	14.31	78.8
	M13	Oxidation $[M+NH_4]^+$	$C_{48}H_{74}O_{15}$	890.5023	908.5361	-0.6	8.52	0.6

m/z = mass-to-charge ratio, ppm = parts per million, R.T. = retention time. Relative peak area was calculated as individual compound peak area divided on total peak area (data not shown).
