# Supplementary material for: Identification of the metabolites of ivermectin in humans
Source: Pharmacol Res Perspect. 2021 Jan 26;9(1):e00712. doi: 10.1002/prp2.712 (PMC7836931; doi:10.1002/prp2.712)
Supplement: Supplementary file 3 — Appendix S1 [file PRP2-9-e00712-s003.pdf]

Processing Parameters

Compound Information

Compound name:	Ivermectin B1a
Chemical formula:	C48H74O14
Adduct:	[M+NH4]+
Charge State:	From:1 To: 1
m/z:	892.5417

Structure

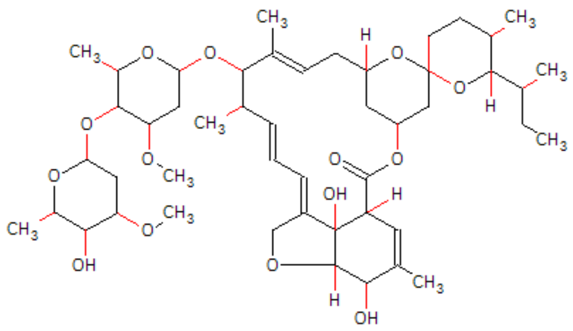

Peak Finding Strategy

The following algorithms were used:

TOF MS

- Predicted metabolites
- Generic peak finding
- Mass defect

TOF MSMS

- Characteristic product ions
  - Find at least 1 ions

Generic Parameters

Biotransformations (Set used: Phase I and II)

Name	Mass Shift	Description
Glucose Conjugation	162.0528	R-OH to R-O-C6H11O5
Demethylation	-14.0157	R-CH3 to R-H
Loss of Water	-18.0106	R-H2O to R
Methylation	14.0157	R-H to R-CH3
Cysteine Conjugation	103.0092	R-COOH to R-CONH-CHCH2SH-COOH
Glutamine Conjugation	128.0586	R-COOH to R-CONHCH((CH2)2CONH2)COOH
Sulfate Conjugation	79.9568	R-OH to R-OSO3H
Taurine Conjugation	107.0041	R-COOH to R-CONH-CH2CH2SO3H
Thioalcohol to Alcohol	-15.9772	RSH to ROH
Glutathione Conjugation	307.0838	+C10H17N3O6S
Demethylation to Carboxylic Acid	29.9742	RCH3 to RCOOH
Oxidation	15.9949	+O
Internal Hydrolysis	18.0106	R-CH=CH-R1 to R-CH2-CHOH-R1
Ketone Formation	13.9793	R-CH2-R1 to R-CO-R1
Demethylation and Oxidation	1.9792	R-CH3 to R-OH
Amine to Carboxylic Acid	28.9789	RNH2 to RCOOH
S-Glutathione Conjugation	305.0682	+C10H15N3O6S
Bis-Ketone Formation	27.9585	R-CH2CH2R1 to R-CO-CO-R1
Oxidative Deamination to Alcohol	0.9840	R-CH2-NH2 to R-CH2-OH

Name	Mass Shift	Description
Oxidation and Ketone Formation	29.9742	+O-CH2+CO
Di-Oxidation	31.9898	+2O
Di-Oxidation and Ketone Formation	45.9691	+2O-CH2+CO
Tri-Oxidation	47.9847	+3O
Tetra-Oxidation	63.9796	+4O
Tetra-Oxidation and Demethylation	49.9640	+4O-CH2
Tri-Oxidation and Demethylation	33.9691	+O3-CH2
Demethylation and Di-Oxidation	17.9742	R-CH2CH3 to R-CH-(OH)2
Deethylation and Carboxylic Acid Formation	3.9585	R-CH2CH3 to R-OOH
N-Acetylation	42.0106	R-NH2 to R-NH-OCCH3
Di-Acetylation of Amines	84.0212	R-NH2 to R-N(COCH3)2
Sulfate and Glucuronide Conjugation	255.9889	+SO3+C6H8O6
Oxidation and Sulfate Conjugation	95.9517	R-H- to R-OSO3H
Di-Oxidation and Taurine Conjugation	138.9939	+2O+C2H7NO3S-H2O
Oxidation and Taurine Conjugation	122.9990	+O+C2H7NO3S-H2O
Oxidation and Sulfate and Glucuronide Conjugation	271.9838	+C6H8O6+O+SO3
Oxidation and Cysteine Conjugation	119.0041	+O+C3H7NO2S-H2O
Phosphorylation	79.9663	R-H to R-H2PO3
Di-Oxidation and Cysteine Conjugation	134.9990	+2O+C3H7NO2S-H2O
Di-Oxidation and Glucuronide Conjugation	208.0219	+2O+C6H8O6
Di-Oxidation and Bis-Glucuronidation	384.0540	2O+2(C6H8O6)
Tri-Oxidation and Glucuronide Conjugation	224.0168	+3O+C6H8O6
Tri-Oxidation and Bis-Glucuronidation	400.0489	3O+2(C6H8O6)
Glycine Conjugation	57.0215	R-COOH to R-CONHCH2COOH
Glutathione Conjugation and Loss of H2S	273.0961	+C10H17N3O6S-H2S
Oxidation and Glutamine Conjugation	144.0535	+O+C5H10N2O3-H2O
Glutathione Conjugation and Oxidation	323.0787	+C10H17N3O6S+O
Hydrolysis of Nitrate Esters	-44.9851	R-ONO2 to R-OH
Decarboxylation	-43.9898	R-COOH to R-H
Propyl Ketone to Acid	-40.0677	R-CH2COC3H7 to R-COOH
Loss of Hydroxymethylene	-30.0106	R-CH2OH to R-H
Nitro Reduction	-29.9742	R-NO2 to R-NH2
Propyl Ether to Acid	-28.0677	R-CH2OCH2CH2CH3 to R-COOH
Loss of CO	-27.9949	R-CO-R1 to R-R1
Ethyl Ketone to Acid	-26.0520	R-CH2COCH2CH3 to R-COOH
Sulfoxide to Thioether	-15.9949	RR1SO to RR1S
Ethyl Ether to Acid	-14.0520	R-CH2OCH2CH3 to R-COOH
Tert-Butyl to Acid	-12.0728	R-C(CH3)3 to R-COOH
Ethyl to Alcohol	-12.0364	R-CH2CH3 to R-OH

Name	Mass Shift	Description
Desaturation	-2.0157	R-CH2-CH2-R1 to R-CHCH-R1
Demethylation and Methylene to Ketone	-0.0364	-CH2-CH2+CO
Oxidation and Internal Hydrolysis	34.0055	+O+H2O
Isopropyl to Acid	1.9429	R-CH(CH3)2 to R-COOH
Hydrogenation	2.0157	+2H
Ethyl to Carboxylic Acid	15.9585	R-CH2CH3 to R-COOH
Oxidation and Methylation	30.0106	R-H to R-OCH3
S-Cysteine Conjugation	119.0041	RR1-CH2 to RR1-CH-SCH2CHNH2-COOH
N-Acetylcysteine Conjugation	161.0147	RR1-CH2 to RR1-CH-SCH2CHNCOCH3-COOH
Bis-Sulfate Conjugation	159.9136	+2(SO3)
Oxidation and Glucuronide Conjugation	192.0270	R-H to R-O-C6H8O6
Bis-Glucuronide Conjugation	352.0642	+2(C6H8O6)
Bis-Demethylation	-28.0313	CH3-R-CH3 to R
Di-Hydrogenation	4.0313	+2H2
Internal Hydrolysis and Di-Oxidation	50.0004	+H2O+2O
Demethylation and Glucuronide Conjugation	162.0165	-CH2 + C6H8O6
Demethylation and Hydrogenation	-12.0000	-CH2 +H2
Oxidation and Bis-Glucuronide Conjugation	368.0591	O+2(C6H8O6)
Bis-Phosphorylation	159.9327	+2(HPO3)
Demethylation and Desaturation	-16.0314	-CH2-H2
Parent	0.0000	Parent (P)
Glucuronidation	176.0321	R-H to R-C6H9O6

Chromatographic Data

Chromatographic Peak

Retention time window: 0 to 18 min

	MS Data	Analog Data
	XIC Width: Automatic	Wavelength (UV only): 190 to 400 nm<br>Time offset from MS: 0 min
LC Peak Separation	25.0%	25.0%
Minimum peak width	2.5 sec.	2.5 sec.
Minimum peak intensity	TOF MS: 2000 cps<br>TOF MSMS: 400 cps	20
Use smoothing	No	No
Sample-control offset	0 min	0 min
Sample/control ratio	3 times greater than control signal	

MS Parameters

m/z Tolerance

MS m/z Tolerance: 10 ppm

Minimum MS peak intensity: 100

Isotope Pattern Tolerances

Intensity tolerance: 20%

MS m/z tolerance: 3mDa

Limits

Maximum number of unexpected metabolites: 100

Generic LC/MS Peak Finding

Perform background subtraction: No

Chosen Adducts

Ion Type	Charge	Radical
[M+H]+	1	False
[M+Na]+	1	False
[M+NH4]+	1	False

MS/MS Parameters

MS/MS Finding

MS/MS m/z tolerance: 5 mDa

Minimum MS/MS peak intensity: 100 cps

MS/MS Isotope Finding

Intensity tolerance: 20 %

MS/MS m/z tolerance: 3 mDa

Source of Reference MS/MS Spectrum: Selected reference spectrum

MS/MS Spectrum

Use advanced MS/MS filter: False

Similarity and Fragment Interpretation

MS/MS m/z tolerance: 5 mDa

Minimum signal-to-noise ratio: 3

Fragment Interpretation Options

Number of fragment peaks selected for assignment: 30

Break aromatic rings: False

Maximum number of bonds to break: 3

Maximum number of C-C bonds to break: 3

Formula Prediction

Search Constraints

Elements from:

Elements to: C68H114N15O34P5S5

Isotope Pattern Tolerance

Intensity tolerance: 10%

MS m/z tolerance: 10 ppm

Ranking

Contribution (from MS to MS/MS data): 30.0%

Automatically weight MS/MS: No

Rings and Double Bonds

RDB from: -1

RDB to: 30

Element Ratios

Oxygen/phosphorus count: >= 2

Oxygen/sulphur count: >= 2

Confirmation Scoring

Property	Maximum Score
Mass defect	100
Isotope pattern	0
MS/MS	100
Mass accuracy	100
Total confirmation score	300

Compound-Specific Parameters

Cleavage Metabolites

Maximum bonds to break: 2

Break ring bonds: True

Only break C-N bonds: False

Selected Cleavage Metabolites: 30

Loss From Parent	Formula	m/z [M+H]+	m/z [M+NH4]+	m/z [M+Na]+
C41H60O11 and CH2O	C6H12O2	117.0910	134.1176	139.0730
C34H48O8 and C7H12O4	C7H14O2	131.1067	148.1332	153.0886
C41H60O10 and CH2O	C6H12O3	133.0859	150.1125	155.0679
C41H60O11	C7H14O3	147.1016	164.1281	169.0835
C41H60O10 and CH2	C6H12O4	149.0808	166.1074	171.0628
C41H60O10	C7H14O4	163.0965	180.1230	185.0784
C34H48O8 and CH2O	C13H24O5	261.1697	278.1962	283.1516
C34H48O8 and O	C14H26O5	275.1853	292.2118	297.1672
C34H48O8 and CH2	C13H24O6	277.1646	294.1911	299.1465
C34H48O8	C14H26O6	291.1802	308.2068	313.1622
C34H48O7 and CH2	C13H24O7	293.1595	310.1860	315.1414
C34H48O7	C14H26O7	307.1751	324.2017	329.1571
C14H24O7 and O	C34H50O6	555.3680	572.3946	577.3500
C14H24O7	C34H50O7	571.3629	588.3895	593.3449
C14H24O6	C34H50O8	587.3578	604.3844	609.3398
CH2O and C7H12O4	C40H60O9	685.4310	702.4576	707.4130
C7H12O4 and O	C41H62O9	699.4467	716.4732	721.4286
CH2O and C7H12O3	C40H60O10	701.4259	718.4525	723.4079
C7H12O4	C41H62O10	715.4416	732.4681	737.4235
CH2 and C7H12O3	C40H60O11	717.4208	734.4474	739.4028
C7H12O3	C41H62O11	731.4365	748.4630	753.4184
CH2O and CH2O	C46H70O12	815.4940	832.5206	837.4759

Loss From Parent	Formula	m/z [M+H]+	m/z [M+NH4]+	m/z [M+Na]+
CH2O and O	C47H72O12	829.5097	846.5362	851.4916
CH2O and CH2	C46H70O13	831.4889	848.5155	853.4709
O and O	C48H74O12	843.5253	860.5519	865.5072
CH2O	C47H72O13	845.5046	862.5311	867.4865
CH2 and CH2	C46H70O14	847.4838	864.5104	869.4658
O	C48H74O13	859.5202	876.5468	881.5022
CH2	C47H72O14	861.4995	878.5260	883.4814
H-2O	C48H76O13	861.5359	878.5624	883.5178

Mass Defect

Name	Formula	*m/z* [M+NH4]+	Defect	Mass Defect Window (mDa)		Mass Range	
				Below	Above	From *m/z*	To *m/z*
Loss of C41H60O11 and CH2O	C6H12O2	134.1176	0.1176	30	20	116	194
Loss of C34H48O8 and C7H12O4	C7H14O2	148.1332	0.1332	30	20	130	208
Loss of C41H60O10 and CH2O	C6H12O3	150.1125	0.1125	30	20	132	210
Loss of C41H60O11	C7H14O3	164.1281	0.1281	30	20	146	224
Loss of C41H60O10 and CH2	C6H12O4	166.1074	0.1074	30	20	148	226
Loss of C41H60O10	C7H14O4	180.1230	0.1230	30	20	162	240
Loss of C34H48O8 and CH2O	C13H24O5	278.1962	0.1962	30	20	260	338
Loss of C34H48O8 and O	C14H26O5	292.2118	0.2118	30	20	274	352
Loss of C34H48O8 and CH2	C13H24O6	294.1911	0.1911	30	20	276	354
Loss of C34H48O8	C14H26O6	308.2068	0.2068	30	20	290	368
Loss of C34H48O7 and CH2	C13H24O7	310.1860	0.1860	30	20	292	370
Loss of C34H48O7	C14H26O7	324.2017	0.2017	30	20	306	384
Loss of C14H24O7 and O	C34H50O6	572.3946	0.3946	30	20	554	632
Loss of C14H24O7	C34H50O7	588.3895	0.3895	30	20	570	648
Loss of C14H24O6	C34H50O8	604.3844	0.3844	30	20	586	664
Loss of CH2O and C7H12O4	C40H60O9	702.4576	0.4576	30	20	684	762
Loss of C7H12O4 and O	C41H62O9	716.4732	0.4732	30	20	698	776
Loss of CH2O and C7H12O3	C40H60O10	718.4525	0.4525	30	20	700	778
Loss of C7H12O4	C41H62O10	732.4681	0.4681	30	20	714	792
Loss of CH2 and C7H12O3	C40H60O11	734.4474	0.4474	30	20	716	794
Loss of C7H12O3	C41H62O11	748.4630	0.4630	30	20	730	808
Loss of CH2O and CH2O	C46H70O12	832.5206	0.5206	30	20	815	893
Loss of CH2O and O	C47H72O12	846.5362	0.5362	30	20	829	907
Loss of CH2O and CH2	C46H70O13	848.5155	0.5155	30	20	831	909

Name	Formula	*m/z* [M+NH4]+	Defect	Mass Defect Window (mDa)		Mass Range	
				Below	Above	From *m/z*	To *m/z*
Loss of O and O	C48H74O12	860.5519	0.5519	30	20	843	921
Loss of CH2O	C47H72O13	862.5311	0.5311	30	20	845	923
Loss of CH2 and CH2	C46H70O14	864.5104	0.5104	30	20	847	925
Loss of O	C48H74O13	876.5468	0.5468	30	20	859	937
Loss of CH2	C47H72O14	878.5260	0.5260	30	20	861	939
Loss of H-2O	C48H76O13	878.5624	0.5624	30	20	861	939
Parent	C48H74O14	892.5417	0.5417	30	20	875	953
Sulphate	C48H74O17S	972.4985	0.4985	30	20	954	1032
Glucuronidation	C54H82O20	1068.5738	0.5738	30	20	1051	1129
Glutathione	C58H91N3O20S	1199.6255	0.6255	30	20	1182	1260
Bis-Glucuronidation	C60H90O26	1244.6059	0.6059	30	20	1227	1305

Isotope Pattern

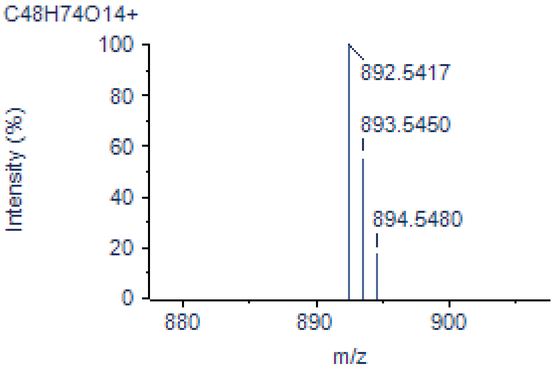

Isotopes

m/z	Intensity (%)	Mass Offset
892.5417	100.0	0.0000
893.5450	55.1	1.0034
894.5480	17.7	2.0063

Intensity tolerance: 20 %

MS *m/z* tolerance: 3 mDa

Product Ions and Neutral Losses

Reference MS/MS spectrum: Compound Library

Add product ions, neutral losses from Phase II metabolites: Yes

Filters

	From	To
**m/z**	50	1000
**Charge State**	1	1

Show only product ions above: 5 %

Mass accuracy within: 5 mDa

m/z	Z	Formula	Error	Neutral Loss	PI	NL	IP
113.0597	1	C6H9O2	0.04	762.45542832	True	False	False
137.0949	1	C9H13O	-1.19	738.4190428	True	False	False
145.0853	1	C7H13O3	-0.67	730.42921352	True	False	False

m/z	Z	Formula	Error	Neutral Loss	PI	NL	IP
162.1118	1	C7H16NO3	-0.66	713.402664392	True	False	False
177.1624	1	C13H21	-1.36	698.35135712	True	False	False
195.1734	1	C13H23O	-0.91	680.3407924	True	False	False
257.139	1	C13H21O5	0.70	618.37678392	True	False	False
307.2266	1	C19H31O3	-0.21	568.2883628	True	False	False
551.3348	1	C34H47O6	-1.96	324.17841824	True	False	False
567.3303	1	C34H47O7	-1.29	308.1835036	True	False	False
569.3452	1	C34H49O7	-2.09	306.16785352	True	False	False
663.3836	1	C32H57NO13	1.18	212.132691352	True	True	False
681.3946	1	C32H59NO14	1.56	194.122126632	True	True	False
695.4125	1	C41H59O9	-2.84	180.09977384	True	True	False
713.426	1	C41H61O10	0.05	162.08920912	True	True	False
				129.042593208	False	True	False
				307.083807344	False	True	False
				176.03208816	False	True	False
				79.95681572	False	True	False
