# Supplementary material for: Identification of the metabolites of ivermectin in humans
Source: Pharmacol Res Perspect. 2021 Jan 26;9(1):e00712. doi: 10.1002/prp2.712 (PMC7836931; doi:10.1002/prp2.712)

Details

Chemical formula: $C_{48}H_{74}O_{14}$

Mass [Da]: 875.09

Solvent: CD3CN

Description:

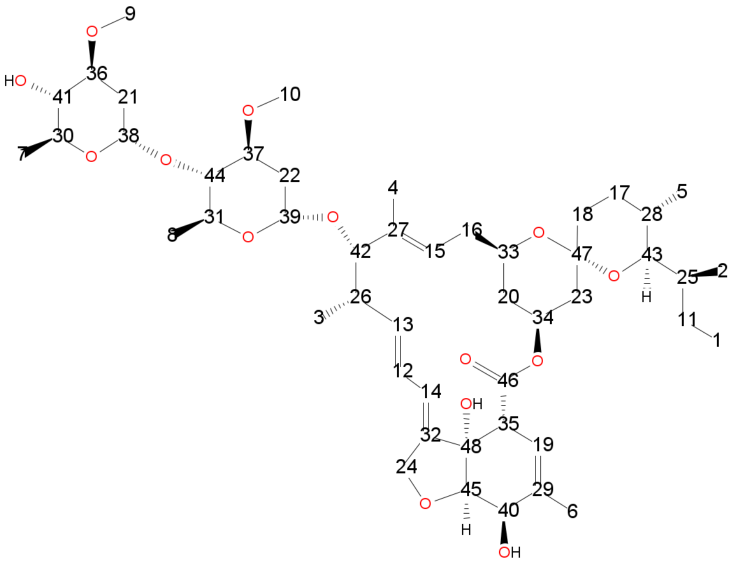

Descriptors

InChI: InChI=1/C48H74O14/
c1-11-25(2)43-28(5)17-18-47(62-43)23-34-20-33(61-47)16-15-27(4)42(26(3)13-12-14-32-24-55-45-40(49)29(6)19-35(46(51)58-34)48(32,45)52)59-39-22-37(

InChIKey: AZSNMRSAGSSBNP-UHFFFAOYNA-N

SMILES: [H]C1(OC2(OC3CC=C(C)C(OC4OC(C)C(OC5OC(C)C(O)C(OC)C5)C(OC)C4)C(C=CC=C6COC7([H])C(O)C(=CC(C(=O)OC(C3)C2)C67O)C)C)CCC1C)C(C)

Project: C:\Data\mago\mg_Mahidol_API_5mm_190603\mg_Mahidol_API_5mm_190605

Report file: C:\Data\mago\mg_Mahidol_API_5mm_190603\mg_Mahidol_API_5mm_190605\mg_Mahidol_API_5mm_190605.pdf

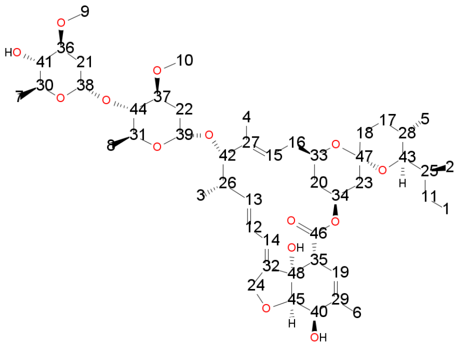

^{1}H table of assignments

Atom	Shift [ppm]	Multiplicity	Bound to	Correlation table
20'	0.79		20 (C32)	H49
5	0.81		5 (C45)	H48
2	0.89		2 (C48)	H47
1	0.97		1 (C47)	H46
3	1.16		3 (C41)	H45
7	1.2		7 (C44)	H44
8	1.23		8 (C43)	H43
23'	1.25		23 (C30)	H42
11'	1.4		11 (C40)	H41
21'	1.46		21 (C35)	H40
22'	1.51		22 (C36)	H39
18'	1.52		18 (C33)	H38
4	1.53		4 (C46)	H36
17	1.53		17 (C39)	H37
28	1.54		28 (C38)	H35
11	1.56		11 (C40)	H34
18	1.61		18 (C33)	H33
25	1.62		25 (C34)	H32
6	1.81		6 (C42)	H31
20	1.9		20 (C32)	H30
23	2.09		23 (C30)	H29
16'	2.24		16 (C37)	H28
21	2.26		21 (C35)	H27
22	2.3		22 (C36)	H26
16	2.31		16 (C37)	H25
26	2.63		26 (C31)	H24

^{13}C table of assignments

Atoms assigned to fragments are shown in italic.

Atom	Shift [ppm]	# H's	Correlation table
2	11.69	3	C48
1	11.74	3	C47
4	14.13	3	C46
5	16.72	3	C45
7	17.11	3	C44
8	17.95	3	C43
6	18.79	3	C42
3	19.68	3	C41
11	27.03	2	C40
17	27.99	2	C39
28	31.1	1	C38
16	33.74	2	C37
22	34.31	2	C36
21	34.55	2	C35
25	35.22	1	C34
18	35.5	2	C33
20	36.5	2	C32
26	39.44	1	C31
23	41.46	2	C30
35	45.61	1	C29
10	55.74	3	C28
9	56.09	3	C27
31	67.14	1	C26
33	67.42	1	C25
40	67.47	1	C24
24	67.54	2	C23
30	68.42	1	C22

Atom	Shift [ppm]	Multiplicity	Bound to	Correlation table
41	3.01		41 (C20)	H23
44	3.18		44 (C16)	H21
35	3.18		35 (C29)	H22
43	3.29		43 (C19)	H20
36	3.33		36 (C18)	H19
10	3.37		10 (C28)	H18
9	3.38		9 (C27)	H17
37	3.62		37 (C17)	H16
30	3.66		30 (C22)	H15
33	3.73		33 (C25)	H14
45	3.78		45 (C15)	H13
31	3.83		31 (C26)	H12
42	3.96		42 (C13)	H11
40	4.19		40 (C24)	H10
24	4.6		24 (C23)	H9
39	4.78		39 (C12)	H8
34	5.04		34 (C21)	H7
15	5.21		15 (C8)	H6
38	5.34		38 (C10)	H5
19	5.42		19 (C9)	H4
13	5.81		13 (C3)	H3
14	5.84		14 (C7)	H2
12	5.89		12 (C6)	H1

Atom	Shift [ppm]	# H's	Correlation table
34	68.55	1	C21
41	76.01	1	C20
43	76.19	1	C19
36	78.05	1	C18
37	79.34	1	C17
44	80.13	1	C16
45	80.37	1	C15
48	80.48	0	C14
42	81.74	1	C13
39	94.89	1	C12
47	97.43	0	C11
38	98.04	1	C10
19	118.65	1	C9
15	118.89	1	C8
14	120.29	1	C7
12	125.16	1	C6
27	134.84	0	C5
29	136.5	0	C4
13	137.39	1	C3
32	140.36	0	C2
46	171.91	0	C1

¹H table of assignments

Atom	Shift [ppm]	Multiplicity	Bound to	Correlation table
20'	0.79		20 (C32)	H49
5	0.81		5 (C45)	H48
2	0.89		2 (C48)	H47
1	0.97		1 (C47)	H46
3	1.16		3 (C41)	H45
7	1.2		7 (C44)	H44
8	1.23		8 (C43)	H43
23'	1.25		23 (C30)	H42
11'	1.4		11 (C40)	H41
21'	1.46		21 (C35)	H40
22'	1.51		22 (C36)	H39
18'	1.52		18 (C33)	H38
4	1.53		4 (C46)	H36
17	1.53		17 (C39)	H37
28	1.54		28 (C38)	H35
11	1.56		11 (C40)	H34
18	1.61		18 (C33)	H33
25	1.62		25 (C34)	H32
6	1.81		6 (C42)	H31
20	1.9		20 (C32)	H30
23	2.09		23 (C30)	H29
16'	2.24		16 (C37)	H28
21	2.26		21 (C35)	H27
22	2.3		22 (C36)	H26
16	2.31		16 (C37)	H25
26	2.63		26 (C31)	H24
41	3.01		41 (C20)	H23

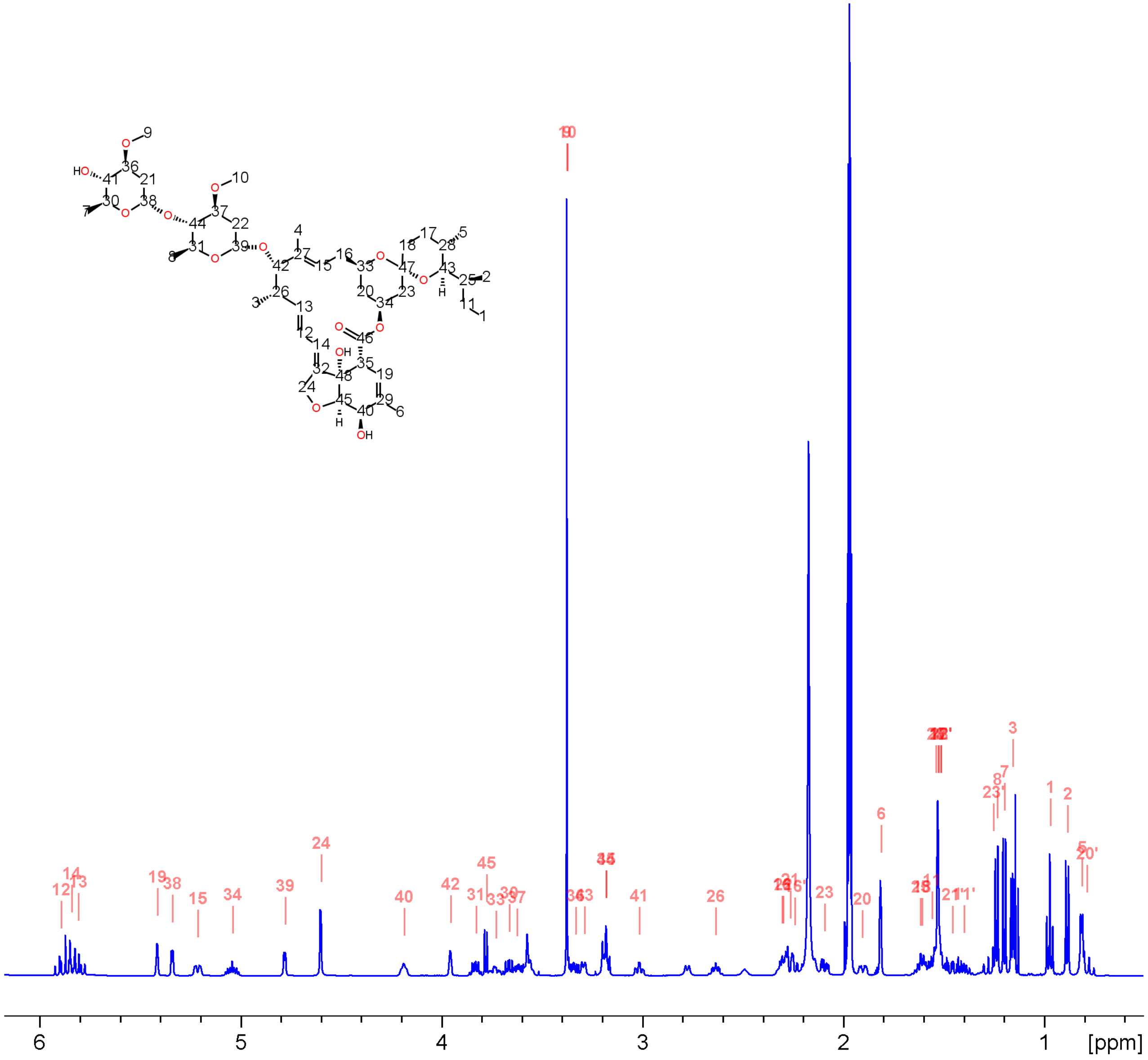

Atom	Shift [ppm]	Multiplicity	Bound to	Correlation table
44	3.18		44 (C16)	H21
35	3.18		35 (C29)	H22
43	3.29		43 (C19)	H20
36	3.33		36 (C18)	H19
10	3.37		10 (C28)	H18
9	3.38		9 (C27)	H17
37	3.62		37 (C17)	H16
30	3.66		30 (C22)	H15
33	3.73		33 (C25)	H14
45	3.78		45 (C15)	H13
31	3.83		31 (C26)	H12
42	3.96		42 (C13)	H11
40	4.19		40 (C24)	H10
24	4.6		24 (C23)	H9
39	4.78		39 (C12)	H8
34	5.04		34 (C21)	H7
15	5.21		15 (C8)	H6
38	5.34		38 (C10)	H5
19	5.42		19 (C9)	H4
13	5.81		13 (C3)	H3
14	5.84		14 (C7)	H2
12	5.89		12 (C6)	H1

^{13}C table of assignments

Atoms assigned to fragments are shown in itallic.

Atom	Shift [ppm]	# H's	Correlation table
2	11.69	3	C48
1	11.74	3	C47
4	14.13	3	C46
5	16.72	3	C45
7	17.11	3	C44
8	17.95	3	C43
6	18.79	3	C42
3	19.68	3	C41
11	27.03	2	C40
17	27.99	2	C39
28	31.1	1	C38
16	33.74	2	C37
22	34.31	2	C36
21	34.55	2	C35
25	35.22	1	C34
18	35.5	2	C33
20	36.5	2	C32
26	39.44	1	C31
23	41.46	2	C30
35	45.61	1	C29
10	55.74	3	C28
9	56.09	3	C27
31	67.14	1	C26
33	67.42	1	C25
40	67.47	1	C24
24	67.54	2	C23

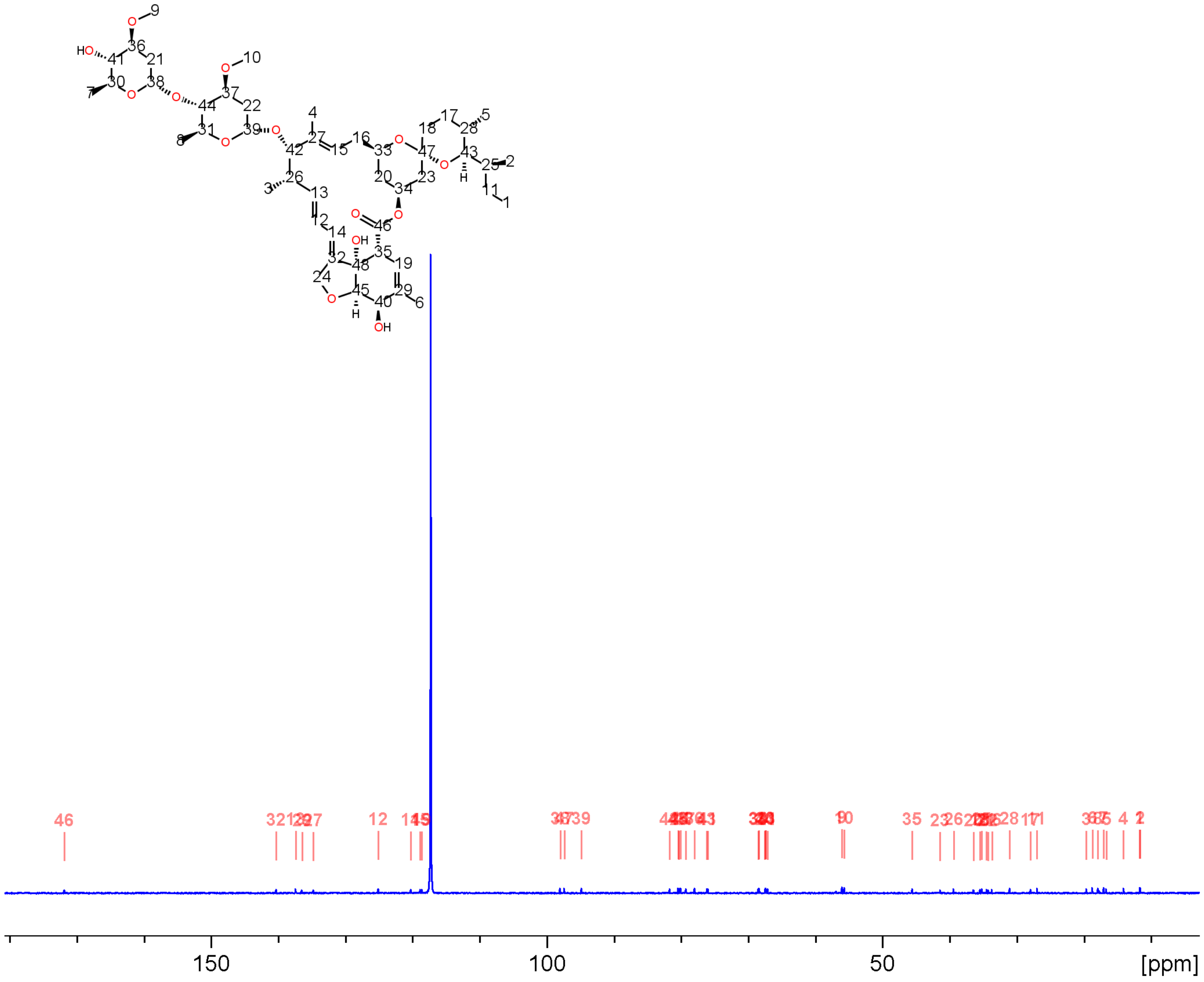

Atom	Shift [ppm]	# H's	Correlation table
30	68.42	1	C22
34	68.55	1	C21
41	76.01	1	C20
43	76.19	1	C19
36	78.05	1	C18
37	79.34	1	C17
44	80.13	1	C16
45	80.37	1	C15
48	80.48	0	C14
42	81.74	1	C13
39	94.89	1	C12
47	97.43	0	C11
38	98.04	1	C10
19	118.65	1	C9
15	118.89	1	C8
14	120.29	1	C7
12	125.16	1	C6
27	134.84	0	C5
29	136.5	0	C4
13	137.39	1	C3
32	140.36	0	C2
46	171.91	0	C1

Explained Correlations

HMBC COSY ROESY

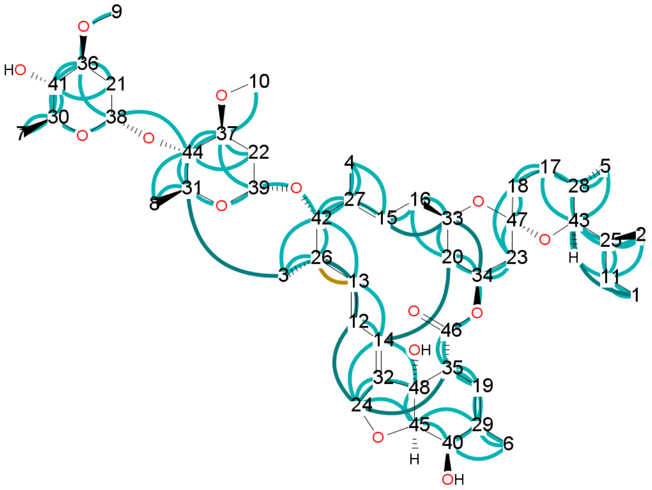

Incorrect Correlations

HMBC

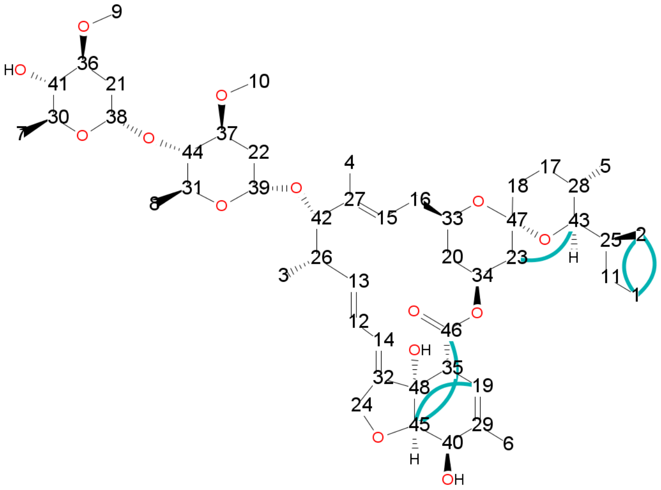

Chemical Shift Correlation

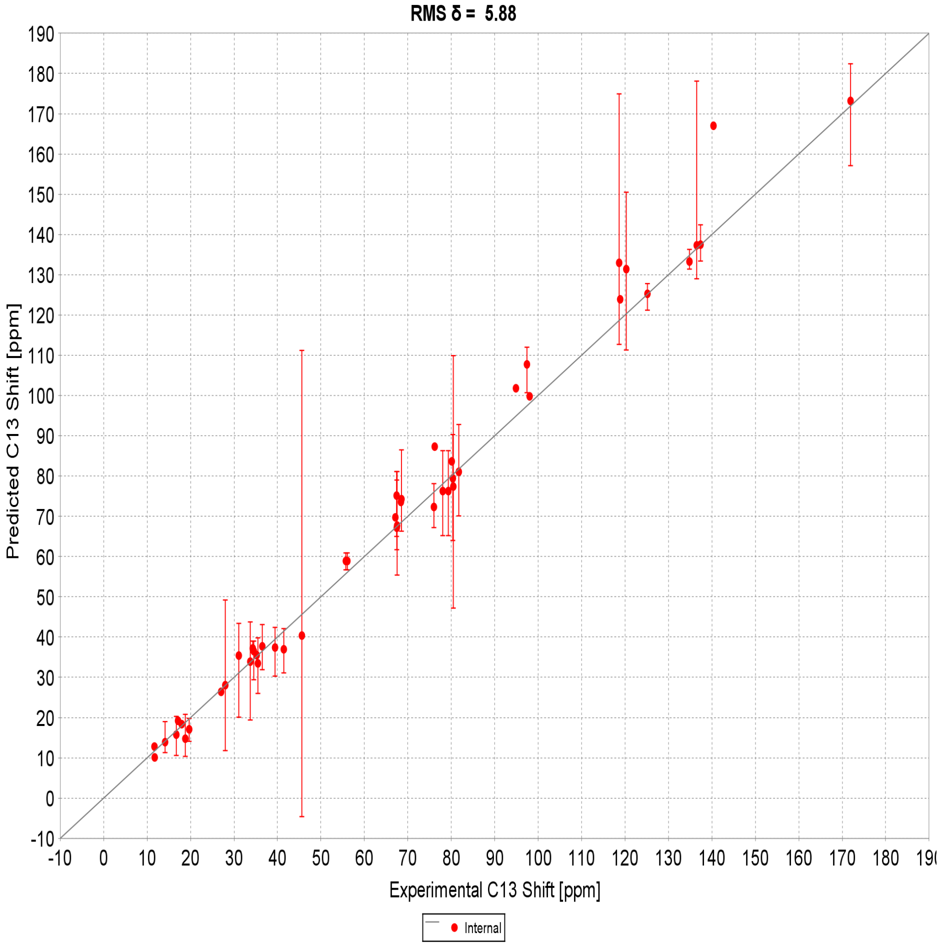

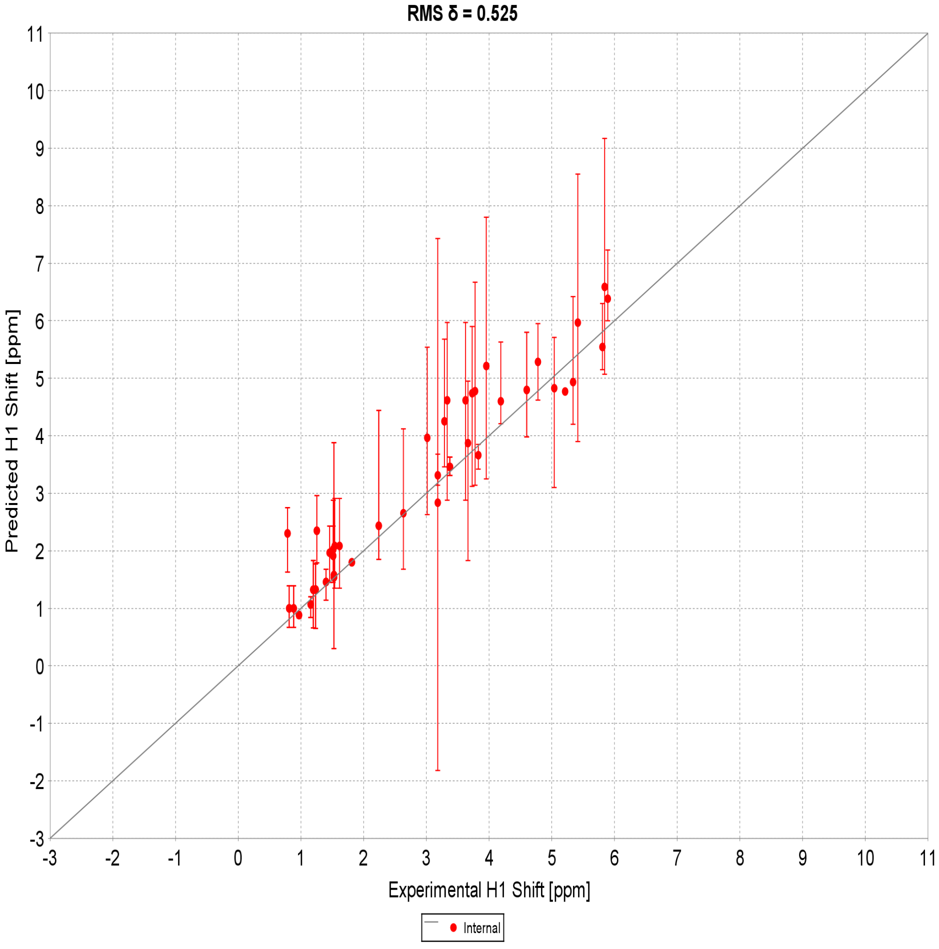

Details

Chemical formula: $C_{47}H_{72}O_{14}$

Mass [Da]: 861.07

Solvent: CD3CN

Description:

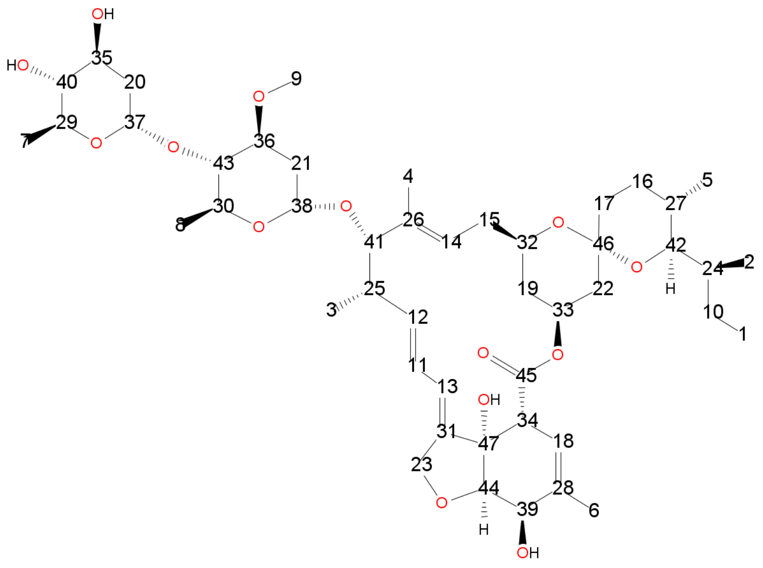

Descriptors

InChI: InChI=1/C47H72O14/
c1-10-24(2)42-27(5)16-17-46(61-42)22-33-19-32(60-46)15-14-26(4)41(58-38-21-36(53-9)43(30(8)56-38)59-37-20-35(48)40(50)29(7)55-37)25(3)12-11-13-31

InChIKey: NMQZPTGPMUJFGY-UHFFFAOYNA-N

SMILES: [H]C1(OC2(OC3CC=C(C)C(OC4OC(C)C(OC5OC(C)C(O)C(O)C5)C(OC)C4)C(C=CC=C6COC7([H])C(O)C(=CC(C(=O)OC(C3)C2)C67O)C)C)CCC1C)C(C)C

Project: D:\mago\mg_Mahidol_mz883_CPTCI800_190816\mg_Mahidol_mz883_CPTCI800_190820

Report file: D:\mago\mg_Mahidol_mz883_CPTCI800_190816\mg_Mahidol_mz883_CPTCI800_190820\mg_Mahidol_mz883_CPTCI800_190820.pdf

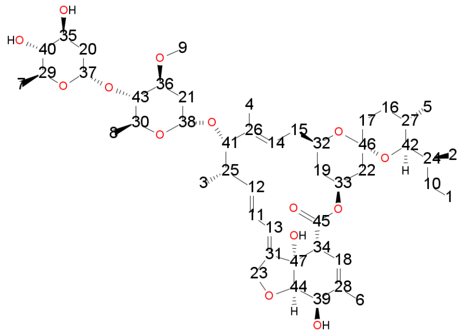

1H table of assignments

Atom	Shift [ppm]	Multiplicity	Bound to	Correlation table
19'	0.8		19 (C32)	H48
5	0.82		5 (C44)	H47
2	0.89		2 (C47)	H46
1	0.97		1 (C46)	H45
3	1.16		3 (C40)	H44
7	1.2		7 (C43)	H43
8	1.24		8 (C42)	H42
22'	1.26		22 (C29)	H41
10'	1.41		10 (C39)	H40
17'	1.52		17 (C33)	H39
16	1.53		16 (C38)	H36
4	1.53		4 (C45)	H37
21'	1.53		21 (C35)	H38
27	1.54		27 (C37)	H35
10	1.56		10 (C39)	H34
20'	1.59		20 (C31)	H33
17	1.6		17 (C33)	H32
24	1.62		24 (C34)	H31
6	1.82		6 (C41)	H30
19	1.91		19 (C32)	H29
20	2.07		20 (C31)	H28
22	2.09		22 (C29)	H27
15'	2.25		15 (C36)	H26
21	2.3		21 (C35)	H25
15	2.31		15 (C36)	H24
25	2.63		25 (C30)	H23

13C table of assignments

Atoms assigned to fragments are shown in italic.

Atom	Shift [ppm]	# H's	Correlation table
2	11.62	3	C47
1	11.65	3	C46
4	14.04	3	C45
5	16.63	3	C44
7	17.01	3	C43
8	17.92	3	C42
6	18.75	3	C41
3	19.62	3	C40
10	27.03	2	C39
16	27.9	2	C38
27	31.07	1	C37
15	33.56	2	C36
21	34.07	2	C35
24	35.17	1	C34
17	35.66	2	C33
19	36.51	2	C32
20	38.02	2	C31
25	39.52	1	C30
22	41.4	2	C29
34	45.67	1	C28
9	55.69	3	C27
30	67.05	1	C26
32	67.4	1	C25
39	67.43	1	C24
23	67.62	2	C23
35	68.43	1	C22
33	68.44	1	C20

Atom	Shift [ppm]	Multiplicity	Bound to	Correlation table
40	2.92		40 (C18)	H22
43	3.18		43 (C16)	H21
34	3.19		34 (C28)	H20
42	3.3		42 (C19)	H19
9	3.37		9 (C27)	H18
36	3.62		36 (C17)	H17
35	3.64		35 (C22)	H16
29	3.67		29 (C21)	H15
32	3.74		32 (C25)	H14
44	3.79		44 (C15)	H13
30	3.83		30 (C26)	H12
41	3.96		41 (C13)	H11
39	4.2		39 (C24)	H10
23	4.61		23 (C23)	H9
38	4.79		38 (C12)	H8
33	5.07		33 (C20)	H7
14	5.21		14 (C8)	H6
37	5.31		37 (C10)	H5
18	5.42		18 (C9)	H4
12	5.8		12 (C3)	H3
11	5.84		11 (C7)	H2
13	5.89		13 (C6)	H1

Atom	Shift [ppm]	# H's	Correlation table
29	68.44	1	C21
42	76.28	1	C19
40	77.7	1	C18
36	79.45	1	C17
43	80.15	1	C16
44	80.31	1	C15
47	80.47	0	C14
41	81.72	1	C13
38	94.81	1	C12
46	97.41	0	C11
37	97.95	1	C10
18	118.6	1	C9
14	118.98	1	C8
11	120.33	1	C7
13	125.15	1	C6
26	134.81	0	C5
28	136.69	0	C4
12	137.56	1	C3
31	140.37	0	C2
45	171.98	0	C1

^{1}H table of assignments

Atom	Shift [ppm]	Multiplicity	Bound to	Correlation table
19'	0.8		19 (C32)	H48
5	0.82		5 (C44)	H47
2	0.89		2 (C47)	H46
1	0.97		1 (C46)	H45
3	1.16		3 (C40)	H44
7	1.2		7 (C43)	H43
8	1.24		8 (C42)	H42
22'	1.26		22 (C29)	H41
10'	1.41		10 (C39)	H40
17'	1.52		17 (C33)	H39
16	1.53		16 (C38)	H36
4	1.53		4 (C45)	H37
21'	1.53		21 (C35)	H38
27	1.54		27 (C37)	H35
10	1.56		10 (C39)	H34
20'	1.59		20 (C31)	H33
17	1.6		17 (C33)	H32
24	1.62		24 (C34)	H31
6	1.82		6 (C41)	H30
19	1.91		19 (C32)	H29
20	2.07		20 (C31)	H28
22	2.09		22 (C29)	H27
15'	2.25		15 (C36)	H26
21	2.3		21 (C35)	H25
15	2.31		15 (C36)	H24
25	2.63		25 (C30)	H23
40	2.92		40 (C18)	H22

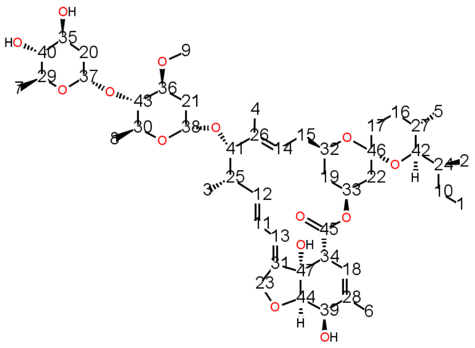

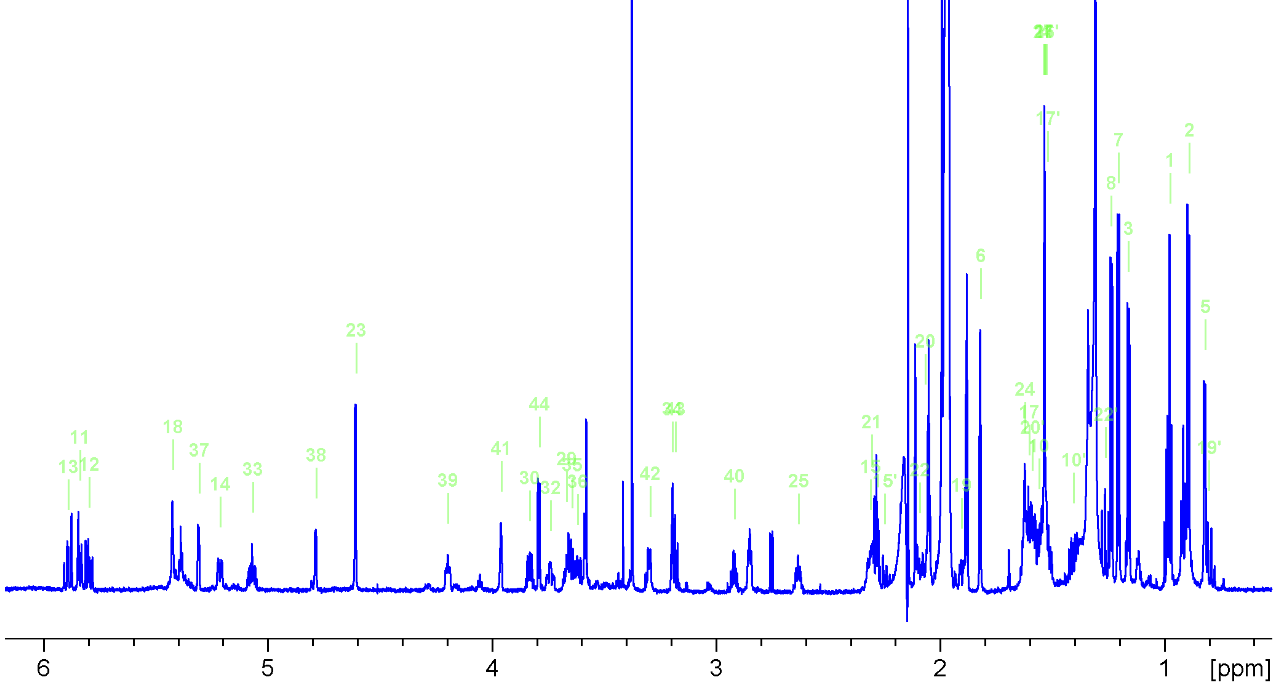

Atom	Shift [ppm]	Multiplicity	Bound to	Correlation table
43	3.18		43 (C16)	H21
34	3.19		34 (C28)	H20
42	3.3		42 (C19)	H19
9	3.37		9 (C27)	H18
36	3.62		36 (C17)	H17
35	3.64		35 (C22)	H16
29	3.67		29 (C21)	H15
32	3.74		32 (C25)	H14
44	3.79		44 (C15)	H13
30	3.83		30 (C26)	H12
41	3.96		41 (C13)	H11
39	4.2		39 (C24)	H10
23	4.61		23 (C23)	H9
38	4.79		38 (C12)	H8
33	5.07		33 (C20)	H7
14	5.21		14 (C8)	H6
37	5.31		37 (C10)	H5
18	5.42		18 (C9)	H4
12	5.8		12 (C3)	H3
11	5.84		11 (C7)	H2
13	5.89		13 (C6)	H1

Explained Correlations

HMBC

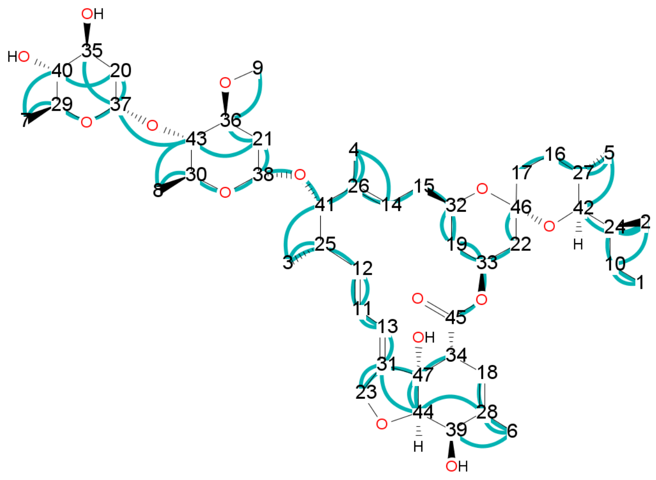

Incorrect Correlations

HMBC

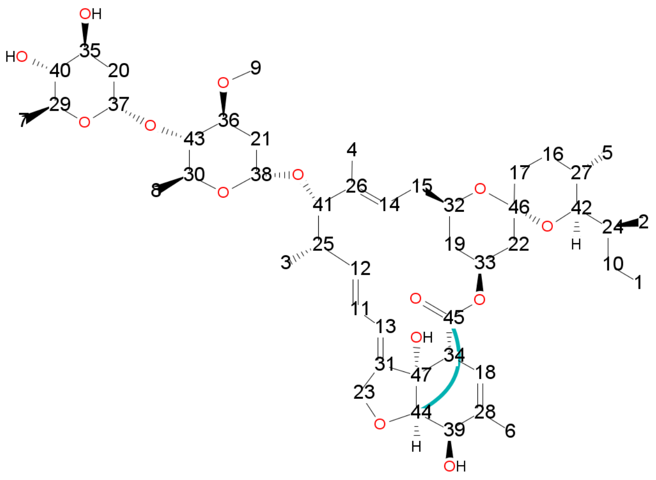

Chemical Shift Correlation

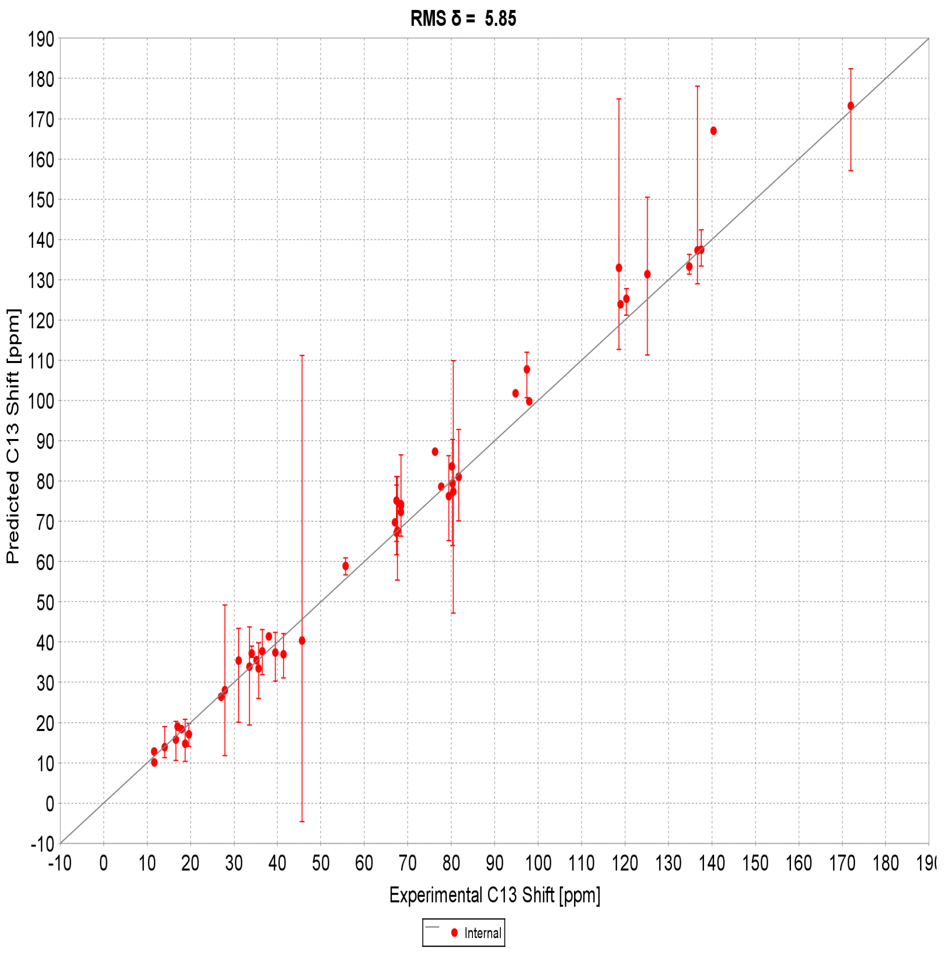

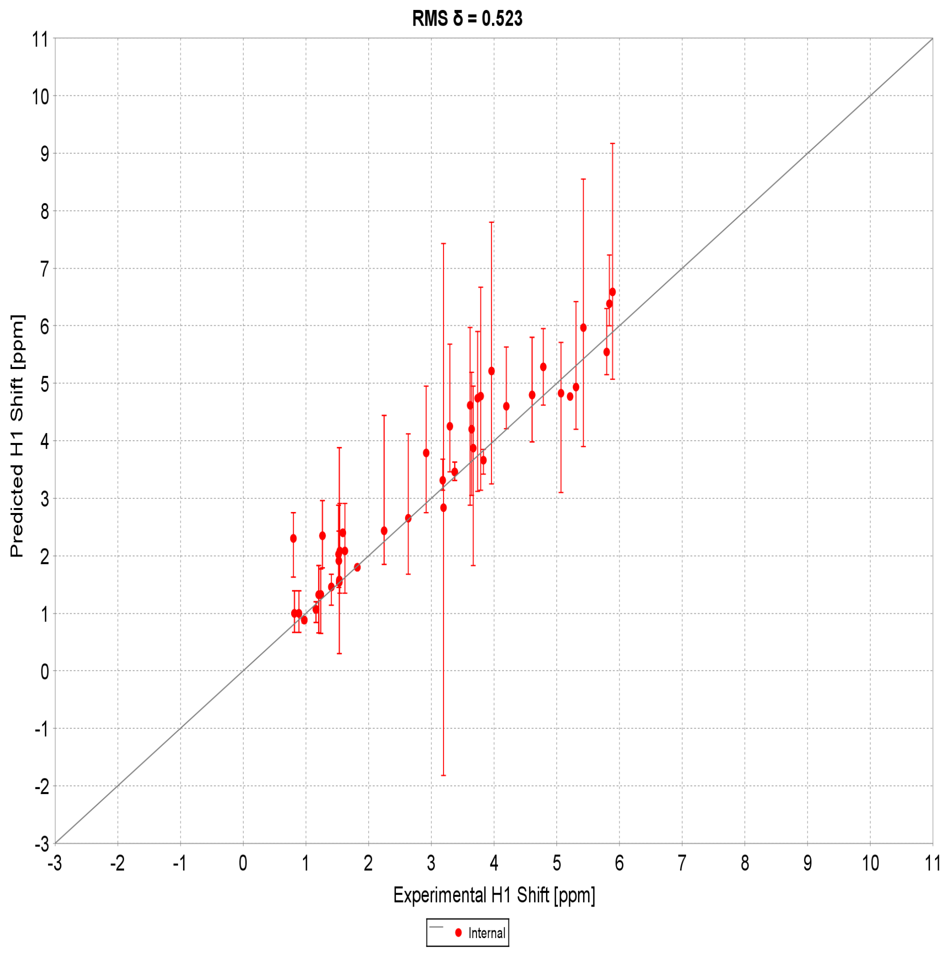

Details

Chemical formula: $C_{48}H_{74}O_{15}$

Mass [Da]: 891.09

Solvent: CD3CN

Description:

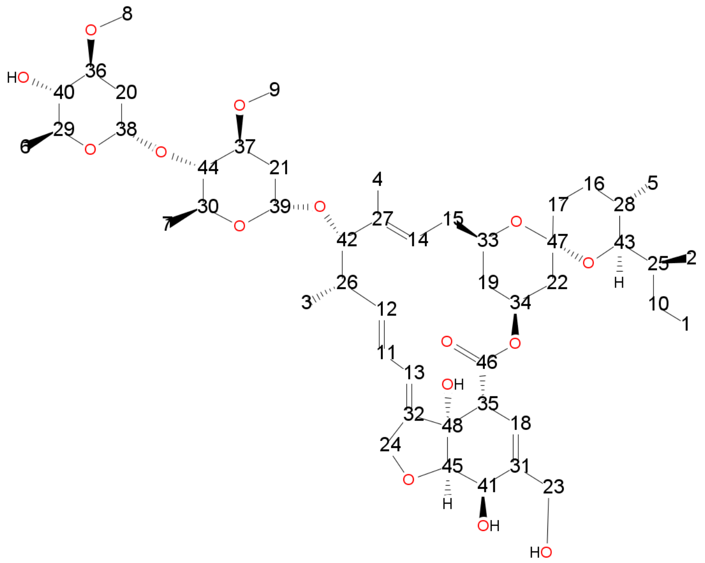

Descriptors

InChI: InChI=1/C48H74O15/
c1-10-25(2)43-28(5)16-17-47(63-43)22-34-19-33(62-47)15-14-27(4)42(60-39-21-37(55-9)44(30(7)58-39)61-38-20-36(54-8)40(50)29(6)57-38)26(3)12-11-13-[illegible]

InChIKey: BGNKRIUWJKFMAF-UHFFFAOYNA-N

SMILES: [H]C1(OC2(OC3CC=C(C)C(OC4OC(C)C(OC5OC(C)C(O)C(OC)C5)C(OC)C4)C(C=CC=C6COC7([H])C(O)C(=CC(C(=O)OC(C3)C2)C67O)CO)C)CCC1C)C(C

Project: D:\mago\mg_Mahidol_mz913_CPTCI800_190809\mg_Mahidol_mz913_800_190814

Report file: D:\mago\mg_Mahidol_mz913_CPTCI800_190809\mg_Mahidol_mz913_800_190814\mg_Mahidol_mz913_800_190814.pdf

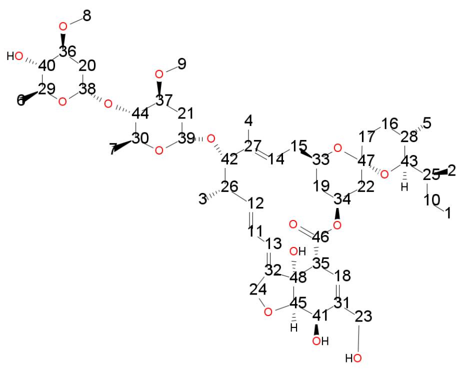

1H table of assignments

Atom	Shift [ppm]	Multiplicity	Bound to	Correlation table
19	0.8		19 (C33)	H49
5	0.82		5 (C45)	H48
2	0.89		2 (C48)	H47
1	0.98		1 (C47)	H46
3	1.17		3 (C42)	H45
6	1.2		6 (C44)	H44
7	1.24		7 (C43)	H43
22'	1.27		22 (C31)	H42
10'	1.41		10 (C41)	H41
20'	1.47		20 (C36)	H40
17'	1.52		17 (C34)	H39
16	1.53		16 (C40)	H37
21	1.53		21 (C37)	H38
28	1.54		28 (C39)	H35
4	1.54		4 (C46)	H36
10	1.57		10 (C41)	H34
17	1.61		17 (C34)	H33
25	1.63		25 (C35)	H32
19'	1.91		19 (C33)	H31
22	2.1		22 (C31)	H30
15'	2.25		15 (C38)	H29
20	2.27		20 (C36)	H28
21'	2.29		21 (C37)	H27
15	2.32		15 (C38)	H26
26	2.64		26 (C32)	H25
40	3.02		40 (C20)	H24

13C table of assignments

Atoms assigned to fragments are shown in italic.

Atom	Shift [ppm]	# H's	Correlation table
2	11.63	3	C48
1	11.67	3	C47
4	14.12	3	C46
5	16.72	3	C45
6	17.08	3	C44
7	17.98	3	C43
3	19.63	3	C42
10	27.01	2	C41
16	27.99	2	C40
28	31.09	1	C39
15	33.68	2	C38
21	34.22	2	C37
20	34.69	2	C36
25	35.22	1	C35
17	35.72	2	C34
19	36.55	2	C33
26	39.55	1	C32
22	41.42	2	C31
35	45.53	1	C30
8	55.57	3	C29
9	56.27	3	C28
23	62.53	2	C27
41	65.22	1	C26
30	67.15	1	C25
33	67.35	1	C24
24	67.66	2	C23
29	68.45	1	C22

Atom	Shift [ppm]	Multiplicity	Bound to	Correlation table
44	3.19		44 (C16)	H23
35	3.25		35 (C30)	H22
43	3.3		43 (C19)	H21
36	3.35		36 (C18)	H20
8	3.38		8 (C29)	H18
9	3.38		9 (C28)	H19
37	3.62		37 (C17)	H17
29	3.68		29 (C22)	H16
33	3.74		33 (C24)	H15
45	3.81		45 (C15)	H14
30	3.84		30 (C25)	H13
42	3.96		42 (C13)	H12
23	4.16		23 (C27)	H11
41	4.41		41 (C26)	H10
24	4.63		24 (C23)	H9
39	4.79		39 (C12)	H8
34	5.09		34 (C21)	H7
14	5.22		14 (C8)	H6
38	5.34		38 (C10)	H5
18	5.7		18 (C9)	H4
12	5.81		12 (C4)	H3
13	5.86		13 (C7)	H2
11	5.9		11 (C6)	H1

Atom	Shift [ppm]	# H's	Correlation table
34	68.6	1	C21
40	76.14	1	C20
43	76.23	1	C19
36	78.06	1	C18
37	79.3	1	C17
44	80.25	1	C16
45	80.48	1	C15
48	80.79	0	C14
42	81.77	1	C13
39	94.9	1	C12
47	97.46	0	C11
38	98.08	1	C10
18	118.64	1	C9
14	118.94	1	C8
13	120.55	1	C7
11	125.18	1	C6
27	134.85	0	C5
12	137.68	1	C4
31	139.78	0	C3
32	140.28	0	C2
46	171.72	0	C1

[1]H table of assignments

Atom	Shift [ppm]	Multiplicity	Bound to	Correlation table
19	0.8		19 (C33)	H49
5	0.82		5 (C45)	H48
2	0.89		2 (C48)	H47
1	0.98		1 (C47)	H46
3	1.17		3 (C42)	H45
6	1.2		6 (C44)	H44
7	1.24		7 (C43)	H43
22'	1.27		22 (C31)	H42
10'	1.41		10 (C41)	H41
20'	1.47		20 (C36)	H40
17'	1.52		17 (C34)	H39
16	1.53		16 (C40)	H37
21	1.53		21 (C37)	H38
28	1.54		28 (C39)	H35
4	1.54		4 (C46)	H36
10	1.57		10 (C41)	H34
17	1.61		17 (C34)	H33
25	1.63		25 (C35)	H32
19'	1.91		19 (C33)	H31
22	2.1		22 (C31)	H30
15'	2.25		15 (C38)	H29
20	2.27		20 (C36)	H28
21'	2.29		21 (C37)	H27
15	2.32		15 (C38)	H26
26	2.64		26 (C32)	H25
40	3.02		40 (C20)	H24
44	3.19		44 (C16)	H23

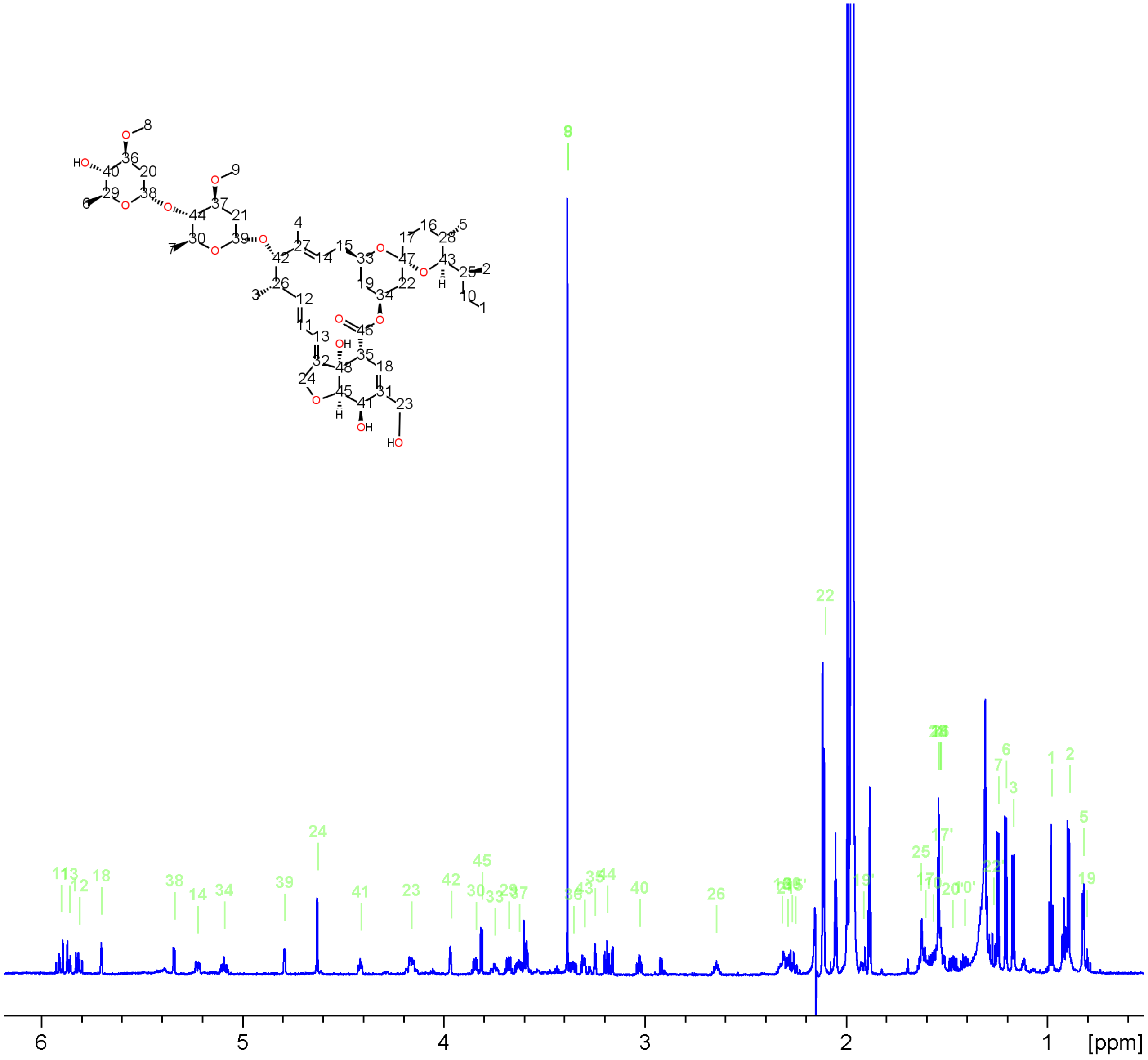

Atom	Shift [ppm]	Multiplicity	Bound to	Correlation table
35	3.25		35 (C30)	H22
43	3.3		43 (C19)	H21
36	3.35		36 (C18)	H20
8	3.38		8 (C29)	H18
9	3.38		9 (C28)	H19
37	3.62		37 (C17)	H17
29	3.68		29 (C22)	H16
33	3.74		33 (C24)	H15
45	3.81		45 (C15)	H14
30	3.84		30 (C25)	H13
42	3.96		42 (C13)	H12
23	4.16		23 (C27)	H11
41	4.41		41 (C26)	H10
24	4.63		24 (C23)	H9
39	4.79		39 (C12)	H8
34	5.09		34 (C21)	H7
14	5.22		14 (C8)	H6
38	5.34		38 (C10)	H5
18	5.7		18 (C9)	H4
12	5.81		12 (C4)	H3
13	5.86		13 (C7)	H2
11	5.9		11 (C6)	H1

Explained Correlations

HMBC

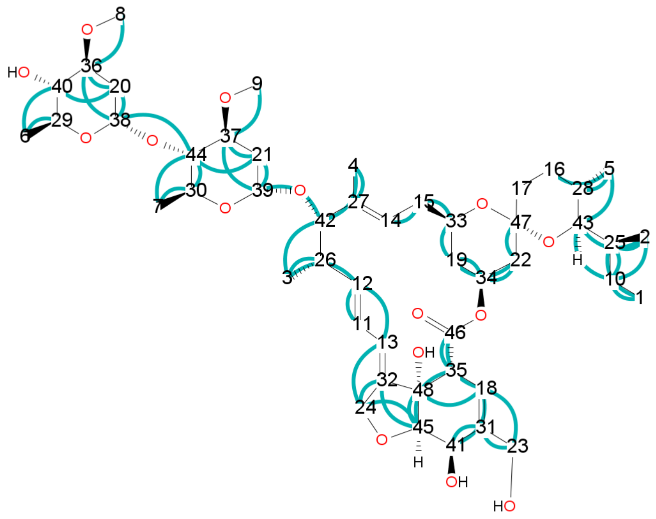

Incorrect Correlations

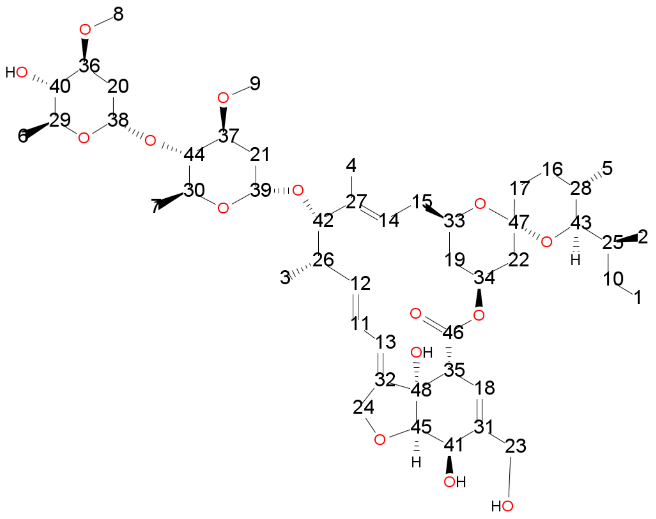

Supplement: Supplementary file 4 — Appendix S2 [file PRP2-9-e00712-s004.pdf]
